# Beyond Δ9-tetrahydrocannabinol and cannabidiol: chemical differentiation of cannabis varieties applying targeted and untargeted analysis

**DOI:** 10.1007/s00216-022-04026-2

**Published:** 2022-04-05

**Authors:** Manuela Carla Monti, Priska Frei, Sophie Weber, Eva Scheurer, Katja Mercer-Chalmers-Bender

**Affiliations:** Institute of Forensic Medicine, Department of Biomedical Engineering, University of Basel, Pestalozzistrasse 22, 4056 Basel, Switzerland

**Keywords:** Principal component analysis, Minor cannabinoids, High-resolution mass spectrometry, Cannabinomics, Metabolomics, Chemotaxonomy

## Abstract

*Cannabis sativa* (*C. sativa*) is commonly chemically classified based on its Δ^9^-tetrahydrocannabinol (THC) and cannabidiol (CBD) content ratios. However, the plant contains nearly 150 additional cannabinoids, referred to as minor cannabinoids. Minor cannabinoids are gaining interest for improved plant and product characterization, e.g., for medical use, and bioanalytical questions in the medico-legal field. This study describes the development and validation of an analytical method for the elucidation of minor cannabinoid fingerprints, employing liquid chromatography coupled to high-resolution mass spectrometry. The method was used to characterize inflorescences from 18 different varieties of *C. sativa*, which were cultivated under the same standardized conditions. Complementing the targeted detection of 15 cannabinoids, untargeted metabolomics employing in silico assisted data analysis was used to detect additional plant ingredients with focus on cannabinoids. Principal component analysis (PCA) was used to evaluate differences between varieties. The overall purpose of this study was to examine the ability of targeted and non-targeted metabolomics using the mentioned techniques to distinguish cannabis varieties from each other by their minor cannabinoid fingerprint. Quantitative determination of targeted cannabinoids already gave valuable information on cannabinoid fingerprints as well as inter- and intra-variety variability of cannabinoid contents. The untargeted workflow led to the detection of 19 additional compounds. PCA of the targeted and untargeted datasets revealed further subgroups extending commonly applied phenotype classification systems of cannabis. This study presents an analytical method for the comprehensive characterization of *C. sativa* varieties.

**Graphical abstract:**

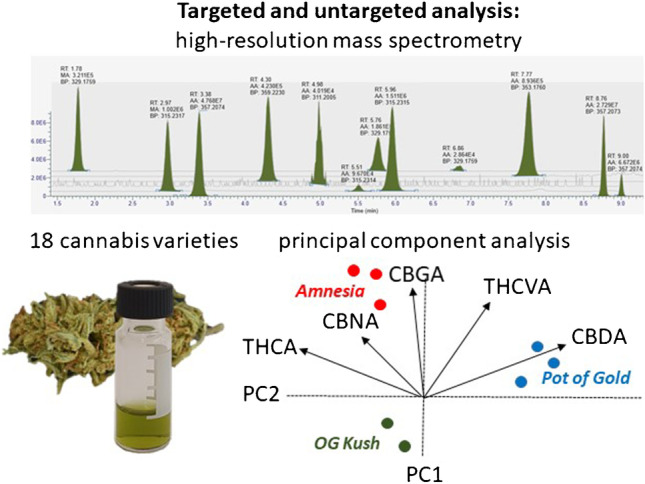

**Supplementary Information:**

The online version contains supplementary material available at 10.1007/s00216-022-04026-2.

## Introduction

*Cannabis sativa* (*C. sativa*) has been cultivated by humans for millennia as a source of fiber (e.g., paper and fabrics), food, and oil. Reports on the medicinal use of *C. sativa* date back to 500 B.C. Arising from the psychoactive effects exerted by Δ^9^-tetrahydrocannabinol (THC), the cannabis plant has a long history of abuse [1]. In recent years, several countries have authorized the dispensing and use of herbal cannabis and cannabis preparations for medical and recreational purposes [2–7]. In 2020, the United Nations reported over 50 countries enrolled in medical cannabis programs and over 15 countries allowing the recreational use of cannabis [8].

*C. sativa* contains hundreds of chemical compounds, of which phytocannabinoids (from here one referred to as cannabinoids) constitute one major class [9]. The best-known cannabinoids are THC and cannabidiol (CBD). In contrast to THC, CBD is regarded as non-intoxicating [10], while exerting various other effects. CBD is, for example, licensed for the treatment of rare forms of childhood epilepsy [11–14]. Even though THC and CBD comprised the main focus of cannabis research so far, nearly 150 additional cannabinoids, often referred to as minor cannabinoids, are known today [15]. The highest cannabinoid concentrations are found in the flowering parts of the female plant [2]. Following a widely accepted [6, 16] chemical classification system that was first introduced in 1973 [17], cannabis phenotypes, also referred to as chemotypes, can be classified based on their content of the two major cannabinoids THC and CBD. Hereby, phenotype I is characterized by THC > CBD (> 0.3% THC, < 0.5% CBD), phenotype II by THC≈CBD (> 0.3 THC, > 0.5% CBD), and phenotype III by THC < CBD (“fiber type,” < 0.3% THC, > 0.5% CBD) [17–19]. Meanwhile, additional phenotypes have been described, with one phenotype presenting cannabigerol (CBG) as major cannabinoid [20]. In the legal context, THC is the main focus of regulatory thresholds, often used to classify a plant or derived product as a narcotic [6, 21]. However, these simple approaches might not be sufficient to characterize a product, which is known to comprise a diversity of bioactive compounds, especially regarding its use as a medicinal product [7]. It is still a subject of ongoing research to what extent pharmacologic effects depend on the chemical profile of a cannabis product. The focus, therefore, shifted from THC and CBD towards more comprehensive approaches with growing interest in the often-overlooked minor cannabinoids, as well as in other compound classes such as flavonoids and terpenoids [7, 18, 22–29].

The growing industry around cannabis and the availability of cannabis products for medicinal and recreational uses necessitates improved product characterization [30, 31] that will enable enhanced product standardization and quality control [31]. The detection of cannabis intake comprises a major task in clinical and forensic toxicology, e.g., in traffic drug testing, abstinence control, and doping control, which is likely to become even more relevant due to increasing medical use and legalization of cannabis products [32]. In forensic toxicology, CBD and minor cannabinoids have been examined as possible markers to distinguish between medicinal and recreational cannabis intake [32–34]. Furthermore, some minor cannabinoids have been investigated as markers for recent cannabis consumption [35, 36] as well as tools to discriminate occasional from frequent consumers [32].

Breeding and selection of *C. sativa* strains resulted in currently over 700 described varieties (also known as cultivars). Even though these varieties might differ in morphologic and organoleptic features and are commonly distinguished by names, it is inconclusive to which extent these varieties present true differences in chemical composition [18]. There are some studies [6, 24, 27, 37–39] addressing this specific question. Fischedick et al. [37] cultivated eleven different varieties under equal and controlled conditions and then analyzed 36 different plant ingredients, seven of which were cannabinoids. Ultimately, the authors were able to distinguish between the investigated varieties. Berman et al. [24] analyzed 36 of the most commonly used cannabis plant varieties prescribed to patients in Israel. They found that despite similar CBD content, not all varieties exerted the same anticonvulsive effect [24], clearly highlighting the need for the determination of further plant ingredients. A recent study conducted by Vasquez-Ocmín et al. [6], which investigated 20 varieties, found minor phytochemicals to play a significant role in the differentiation of *C. sativa* varieties. Cerrato et al. [27] presented an untargeted metabolomics approach, labelled as *phytocannabinomics*, which was tested on 50 cannabis varieties, ultimately proving the existence of chemical subgroups that extend traditional classification systems. Slosse et al. [40] investigated intra- and inter-plantation variabilities by means of chemical fingerprints with the aim of elaborating on common sample sources, e.g., linking seized material to plantations [40]. Finally, Capriotti et al. [30] recently reviewed analytical applications for the characterization of cannabis products applying mass spectrometry. The increasing use of untargeted approaches to achieve better product characterization has been pointed out, while the lack of standardization for untargeted analyses was mentioned as a potential hurdle.

In order to interpret data comprising a large set of analytes, multivariate analyses are commonly used. The aim of such statistical analyses is to identify underlying patterns indicating differences and similarities in the chemical fingerprints. Those patterns would otherwise not be easily recognizable, due to the complexity of the data arising from the large number of analytes (i.e., observations) per sample. Principal component analysis (PCA) describes a mathematical procedure allowing multicomponent data to be reduced in its dimensions. Thereby, PCA enables multidimensional data to be presented in a two-dimensional manner, facilitating data interpretation [41].

On a rudimentary level, the analytical method clearly has an impact on the detectability of cannabis ingredients and, therefore, the knowledge of their composition in the cannabis products [42, 43]. In the cannabis plant, cannabinoids are mainly biosynthesized in their acidic forms, e.g., THC-acid (THCA). These acidic precursors are heat-labile. Chromatographic separation by gas chromatography (GC) typically results in the decarboxylation of cannabinoids in the injection port [24]. In order to investigate acid precursors, high-performance liquid chromatography (HPLC) is preferred [24]. Furthermore, when applying mass spectrometry, the ionization mode substantially influences the ionization efficiency of analytes. While positive ionization mode could be more suitable for the detection of neutral cannabinoids, acidic cannabinoids, which are predominantly found in native plant extracts, are commonly analyzed using negative ionization mode [30]. Therefore, positive and negative ionization modes have been used in the presented work for neutral and acidic cannabinoids, respectively. Finally, the herein used electrospray ionization (ESI) is the most common ionization technique used for HPLC coupled to mass spectrometry [44].

This work reports on the implementation, validation, and application of an analytical method employing HPLC coupled to high-resolution mass spectrometry (HRMS). The method was validated for the quantification of 15 cannabinoids. The application of a full scan acquisition enabled retrospective identification of additional plant ingredients applying an untargeted metabolomics workflow. In-depth cannabinoid fingerprint characterization was conducted for 45 individual plants belonging to 18 cannabis varieties grown under standardized conditions, applying PCA to determine similarities and differences between the investigated varieties. Study aims included the assessment of intra- and inter-variety differences in cannabinoid contents of cannabis plants cultivated and stored under identical conditions.

## Materials and methods

### Materials

Certified reference materials (CRMs) were purchased from Merck (Buchs, Switzerland), Lipomed AG (Arlesheim, Switzerland), or Cayman Chemical Company (MI, USA). Detailed information is found in the supplementary Table S1. LC–MS grade methanol (MeOH), acetonitrile (ACN), and water were purchased from Macherey Nagel (Oensingen, Switzerland). Formic acid (purity 98–100%) was purchased from Merck (Zug, Switzerland). Dried flowers of hops PhEur were purchased from TeeFischer (Tägerwilen, Switzerland); organic peppermint and stinging nettles herbal tea were both purchased from Coop supermarket-chain (Basel, Switzerland).

### Cannabis plant cultivation and harvest

Cannabis inflorescences were kindly provided by Suisse BioHemp AG (Ried bei Kerzers, Switzerland). Cannabis plants were planted in the beginning of July 2020 and harvested by mid-October (98 days). Cultivation took place in a greenhouse of 10,000 m^2^, of which 320 m^2^ were used for the investigated strains. No artificial lighting was applied, temperatures ranged from 10 to 33 °C, and relative humidity ranged from 40 to 75%. Cannabis inflorescences were harvested manually and dried at 38 °C for 36 h until residual water content was 14%. Thereafter, cannabis inflorescences were openly stored at 20 °C and 50% relative humidity in the dark for 2 weeks and finally packaged in separate pressure lock bags, stored in the dark at room temperature until analysis. Samples derived from 45 individual plants belonging to 18 varieties were obtained. A list of all varieties, number of plants per variety, and detailed information on cultivation, i.e., if a plant was grown from seeds or cuttings, are shown in Table 1. Authorization for cultivation and analysis of the herein presented plants and derived samples for research purposes was granted by Swiss regulatory instances.

**Table 1:** Overview of investigated varieties and number of individual plants per variety (*n*). Strains presenting names connected by an “ × ” were obtained via crossbreeding of the respective varieties

	*n*	Cultivation method
Amnesia	1	Cuttings
Amnesia S5^a^	3	Cuttings
Amnesia × SFV	2	Seeds
Big Bud	3	Cuttings
Bubba Kush	3	Cuttings
C7	1	Cuttings
C7 × Thai	1	Cuttings
Durban × Malawi	8	Seeds
GWS	1	Cuttings
Lebi 2	3	Cuttings
Malawi × Super Skunk	1	Cuttings
OG Kush	3	Cuttings
Pot of Gold	2	Cuttings
Pot of Gold nr. 11^b^	1	Cuttings
Purple Punch	3	Cuttings
Rascal OG	3	Cuttings
SFV OG	3	Seeds
Wappa	3	Cuttings

^a^Selection of the Amnesia variety

^b^Selection of the Pot of Gold variety

### Sample extraction

Dried cannabis inflorescences (5 g per individual plant) were homogenized using a Grindomix GM 200 knife mill from Retsch (Haan, Germany). For sample extraction, 50 mg of homogenized cannabis inflorescence were mixed with 2 mL MeOH in glass vials and ultra-sonicated for 15 min. The extract was filtered using a Simplepure™ syringe filter (13 mm, 0.45 µm) obtained from BGB Analytik AG (Boeckten, Switzerland). In a preliminary experiment, the herein applied extraction procedure was evaluated by comparison of the cannabinoid levels obtained after single extraction to a procedure applying an exhaustive extraction comprised of five subsequent extraction steps. The analysis of the combined extract did not result in higher cannabinoid levels compared to the presented protocol (data not shown). Before chromatographic analysis, the extracts were diluted with MeOH to the appropriate concentrations for analysis and calibration range (1:10,000, selected samples were reinjected at 1:5000 or 1:15,000). For each individual plant, extraction was done in duplicate.

### LC-HRMS analysis

Chromatographic separation was achieved using a Dionex UltiMate 3000 UHPLC System equipped with a MultiSLEEVE column heater (Analytical SALES & SERVICES, Inc.), a Triplus RSH Autosampler (CTC Analytics AG), and a Hypersil GOLD™ column (100 × 2.1 mm, 1.9 µm), all purchased from Thermo Fisher Scientific (Reinach, Switzerland). The Autosampler temperature was 10 °C. An injection volume of 5 µL, column temperature of 40 °C, and flow rate of 0.6 mL/min were applied. Mobile phase A consisted of 0.1% (v/v) formic acid in water. Mobile phase B consisted of 50:50% (v/v) ACN and MeOH with 0.1% (v/v) formic acid. The gradient started at 65% of phase B and then increased to 76% over 8.5 min and ramped up to 100% of phase B within the next minute. This condition was maintained for 2 min and followed by 1.5 min reequilibration at starting conditions. For the subsequent analysis, a Q Exactive™ HF mass spectrometer operated with a HESI-II probe all purchased from Thermo Fisher Scientific (Reinach, Switzerland) was used. Transfer capillary temperature was set to 300 °C, spray voltage was set to 3.5 kV, sheath gas flow rate was set to 50 arbitrary units (AU), auxiliary gas flow rate was set to 15 AU, and auxiliary gas heater temperature was 350 °C.

A full scan acquisition over a range of 250–400 m*/z* was performed at a resolution of 120,000 at full width at half maximum (FWHM). To be able to measure at high mass-resolution (> 100,000 FWHM) while maintaining a reasonable cycle time and, thus, sufficient data points per peak, the positive and negative ionization modes were defined in two separate instrument methods, requiring two injections per sample. Maximum injection time (IT) was set to 200 ms. Automatic gain control (AGC) target values of 1e6 and 1e5 were used for the positive and negative ionization modes, respectively. Instruments were controlled and data were processed employing Aria MX, TraceFinder (version 4.1), and FreeStyle™ (version 1.7 SP1) all by Thermo Fisher Scientific (Reinach, Switzerland). To prevent carry-over, blank injections (100% MeOH) were interposed in-between analyses of each plant.

### Quantification of targeted cannabinoids

An overview of the 15 quantified analytes and abbreviations, applied calibration ranges, including weighing factors, referenced internal standards (ISTDs), screened theoretical mass traces, ionization modes, and retention times is given in Table 2. Structures of the targeted cannabinoids are shown in supplementary Table S2. Exemplary chromatograms obtained after injection of quality control (QC) samples are presented in supplementary Fig. S1. QC samples and calibrators were independently from each other generated by dilution of CRM in MeOH. QC samples and calibrators were prepared from separate pooled stock solutions (10 µg/mL in MeOH, stored at − 20 °C) containing either all analytes measured in positive or negative ionization mode. ISTDs were added to calibrators, QC samples, and extracted inflorescences at 100 ng/mL and 500 ng/mL (only THC-COOH) final concentration during the final dilution step. The calibration range for all cannabinoids measured in positive and negative ionization modes, except for THCA and CBDA, was defined from 0.5 to 100 ng/mL. THCA and CBDA were quantified using two separate calibration ranges: 0.5–100 ng/mL (THCA_low_, CBDA_low_) referenced to THC-COOH-D_9_ at 100 ng/mL; 50–500 ng/mL (THCA_high_, CBDA_high_) referenced to the ISTD THC-COOH at 500 ng/mL. Signals falling in between 50 and 100 ng/mL were calculated using the THCA_low_ and CBDA_low_ calibration ranges. For quantification, the analytes were identified via their retention time with a detection window of ± 30 s as well as acceptable mass error ± 5 ppm. During each sequence, QC samples spanning the calibration range (0.8 ng/mL, 3 ng/mL, 80 ng/mL, for all analytes, THCA and CBDA additionally: 425 ng/mL) were measured in order to assure functionality of the analysis, accurate retention times and suitability, and correctness of calibration. For each individual plant, mean values of analyses of the duplicate extractions were used to describe the cannabinoid content.

**Table 2:** Overview of the retention times, chemical formula, measured polarity and respective [M + H]^+^ or [M-H]^−^ signals, calibration ranges, weighing of the calibration curve, and internal standards (ISTDs) used for the quantitative analysis. Cannabinoids are ordered based on retention time (RT). THC-OH-D_3_: deuterated 11-hydroxy-THC (human THC metabolite); THC-COOH and THC-COOH-D_9_: (deuterated) 11-nor-9-carboxy-THC (human THC metabolite)

Cannabinoid	Abbreviation	RT	Formula	Mass trace [*m*/*z*]		Calibration range [ng/mL]	Weighing of calibration curve	ISTD
Cannabidivarin	CBDV	1.30	C_19_H_26_O_2_	[M + H]^+^	287.2006	0.5–100	1/x	OH-THC-D_3_
Cannabidivarinic acid	CBDVA	1.75	C_20_H_26_O_4_	[M-H]^−^	329.1758	0.5–100	1/x	THC-COOH-D_9_
Cannabidiol	CBD	3.00	C_21_H_30_O_2_	[M + H]^+^	315.2319	0.5–100	1/x	CBD-D_3_
Tetrahydrocannabivarin	THCV	3.10	C_19_H_26_O_2_	[M + H]^+^	287.2006	0.5–100	1/x	THC-D_3_
Cannabigerol	CBG	3.20	C_21_H_32_O_2_	[M + H]^+^	317.2475	0.5–100	1/x	CBD-D_3_
Cannabidiolic acid	CBDA_low_	3.40	C_22_H_30_O_4_	[M-H]^−^	357.2071	0.5–100	1/x	THC-COOH-D_9_
	CBDA_high_	3.40	C_22_H_30_O_4_	[M-H]^−^	357.2071	50–500	1/x	THC-COOH
Cannabigerolic acid	CBGA	4.25	C_22_H_32_O_4_	[M-H]^−^	359.2228	0.5–100	1/x	THC-COOH-D_9_
Cannabinol	CBN	5.00	C_21_H_26_O_2_	[M + H]^+^	311.2006	0.5–100	1/x	CBN-D_3_
Tetrahydrocannabivarinic acid	THCVA	5.71	C_20_H_26_O_4_	[M-H]^−^	329.1758	0.5–100	1/x	THC-COOH-D_9_
Tetrahydrocannabinol	THC	5.90	C_21_H_30_O_2_	[M + H]^+^	315.2319	0.5–100	1/x	THC-D_3_
Cannabicyclol	CBL	6.65	C_21_H_30_O_2_	[M + H]^+^	315.2319	0.5–100	1/x	THC-D_3_
Cannabinolic acid	CBNA	7.66	C_22_H_26_O_4_	[M + H]^+^	353.1758	0.5–100	1/x	THC-COOH-D_9_
Cannabichromene	CBC	7.95	C_21_H_30_O_2_	[M + H]^+^	315.2319	0.5–100	1/x	CBC-D_9_
Tetrahydrocannabinolic acid	THCA_low_	8.75	C_22_H_30_O_4_	[M-H]^−^	357.2071	0.5–100	1/x	THC-COOH-D_9_
	THCA_high_	8.75	C_22_H_30_O_4_	[M-H]^−^	357.2071	50–500	None	THC-COOH
Cannabichromenic acid	CBCA	9.05	C_22_H_30_O_4_	[M-H]^−^	357.2071	0.5–100	1/x	THC-COOH-D_9_
ISTD								
THC-OH-D_3_		2.12	C_21_H_27_O_3_D_3_	[M + H]^+^	334.2456	100		
THC-COOH-D_9_		2.70	C_21_H_19_O_4_D_9_	[M-H]^−^	352.2480	100		
THC-COOH		2.70	C_21_H_28_O_4_	[M-H]^−^	343.1915	500		
CBD-D_3_		3.00	C_21_H_27_O_2_D_3_	[M + H]^+^	318.2507	100		
CBN-D_3_		5.00	C_21_H_23_O_2_D_3_	[M + H]^+^	314.2194	100		
THC-D_3_		5.90	C_21_H_27_O_2_D_3_	[M + H]^+^	318.2507	100		
CBC-D_9_		7.95	C_21_H_21_O_2_D_9_	[M + H]^+^	324.2883	100		

Selectivity and specificity were evaluated by investigating interfering signals in diluted extracts of dried flowers of hops, dried peppermint leaves, and dried stinging nettles as well as blank measurements, with and without the addition of ISTDs. Additionally, Δ8-THC was injected to investigate the separation power between Δ9-THC (here referred to as THC) and its isomer Δ8-THC. Limits of detection (LODs) were investigated after serial dilution of CRM at ranges at suspected LODs. The required root mean square signal to noise (S/N) ratio at the LOD was defined to be ≥ 3. For the evaluation of LOQs, five repeated measurements of the target analytes at 0.5 ng/mL were conducted, followed by evaluation of bias and repeatability as relative standard deviation (RSD), whereby bias within ± 20% and RSD ≤ 20% were considered acceptable. Linearity was tested by measurement of the calibration curves and assessment of the resulting coefficients of correlation (*R*^2^) with a resulting value of > 0.99 regarded sufficient. Accuracy with precision and trueness was evaluated by duplicate measurements of QC samples at different concentration levels on eight different days (0.8 ng/mL, 3 ng/mL, 80 ng/mL, CBDA and THCA additionally: 400 ng/mL). Intra- and inter‐day precision (RSD_r_ and RSD_(T)_) and trueness (as bias) were examined, with validation criteria being RSD_r_ and RSD_(T)_ < 20% and bias within ± 20%.

### Untargeted screening

The high-resolution full scan measurement enabled the retrospective analysis of chromatograms regarding initially untargeted, additional compounds. Due to the overall higher abundancy of acidic cannabinoids in native plant extracts, untargeted data analysis in negative ionization mode yielded more promising results regarding the number of detected compounds and signal intensities, than seen in a preliminary analysis conducted for the positive ionization mode (data not shown). Thus, the untargeted workflow was conducted for the negative ionization mode only. A so-called *unexpected workflow* (predefined workflow within the used software) was adapted in the Compound Discoverer™ (version 3.1.0.305) software from Thermo Fisher Scientific (Reinach, Switzerland). The full scan data was investigated applying an untargeted metabolomics workflow, in which retention times were aligned between samples, mass traces detected, background compounds extracted (comparison to a blank injection), and initially targeted compounds, of where CRMs were available, were detected based on a mass list containing the corresponding retention times and molecular formulas. Supplementary Fig. S2 depicts the complete workflow including advanced parameters used for data processing in the Compounds Discoverer™ software. In a second step, the processed and visualized results for tentatively identified compounds were manually validated. Signals likely corresponding to cannabinoids or other additional plant metabolites were marked for further evaluation and finally exported by means of an inclusion list for additional structure elucidation.

### Structure elucidation of additional compounds

For further characterization of additional compounds, selected samples containing the compounds of interest were reinjected applying a full scan measurement with a data-dependent-MS^2^ (dd-MS^2^) acquisition. The resolution of the full scan measurement was 120,000 FWHM, with AGC target value of 1e6 and maximum IT of 200 ms. Method parameters for the dd-MS^2^ acquisition were resolution of 30,000, AGC target value of 1e5, maximum IT of 20 ms, and isolation window of 2.0 m*/z*. Suitable collision energy (CE) was determined in a preliminary experiment via measurement of CBDA, THCA, and CBCA at 100 ng/mL applying varying CEs (20, 30, 40, 50, 60), after which CE 40 was chosen as best (data not shown). Tentatively identified compounds were compared to literature based on proposed elemental composition derived from the [M-H]^−^ signal and MS^2^ spectra.

### Multivariate analyses

PCA was conducted in R (version 3.4.3). Source codes for analyses conducted in R are presented in supplementary Figs. S3 and S4. Statistical analyses were conducted for the results of the targeted analysis (mass content) as well as for exported and weight normalized mean peak areas (exported from the Compound Discoverer™ software) for the untargeted approach. PCA analysis using the R package FactoMineR [45] included data normalization (z-transformations; autoscaling) as a data pretreatment, meaning that the result of each analyte (i.e., observation; content w/w or weight normalized peak area) is mean-centered and divided by its standard deviation. Ultimately, this results in a mean value equaling zero and a standard deviation of one. Scatter plots, generated by plotting PC1 against PC2, offered the possibility to assess similarities and differences between varieties (plotted as individual data points per plant). If varieties show up close to each other, this indicates a high degree of similarity, if they spread apart, this means that these varieties are considerably different regarding their chemical composition. The contribution of individual analytes is made visible by additionally plotting their corresponding eigenvectors (e.g., biplots, loading plots). Thereby, the direction and length of an eigenvector represent its contribution to the construction of the dimensions (PC1 and PC2), allowing to identify which analytes are contributing the most. Analytes that largely add to a dimension are interesting, as they are acting as distinguishing markers between varieties. Detailed information concerning PCA in general [41] and specifically the applied package [45] are found under the indicated literature sources. Complementing the PCA additionally, heatmaps applying hierarchical clustering of the z-transformed data were computed in R using the ggplot2 package. These heatmaps allow a complementary representation of the data.

## Results

### HPLC-HRMS analysis and method validation

Selectivity and specificity of the method were shown by analysis of tea extracts, solvent blanks, and solvent blanks containing ISTDs, as no signals were detected in the defined time frames and corresponding mass traces of the targeted analytes. With the presented method THC (Δ9-THC) and its isomer Δ8-THC are chromatographically separated. However, due to the close elution of Δ8-THC which ultimately coelutes within the tail of the THC peak, full quantification of Δ8-THC, which is expected to occur at much lower levels compared to THC [24], was omitted. The LOD for Δ8-THC was determined to be 5 ng/mL if 500 ng/mL THC was contained in a spiked sample, corresponding to 0.2% Δ8-THC and 20% THC (w/w; 1:10,000 dilution when 50 mg plant material are extracted with 2 mL MeOH). An exemplary chromatogram is shown in supplementary Fig. S5. For all analytes measured in negative ionization mode, an LOD of 0.2 ng/mL was observed, translating to cannabinoid contents at product level of 0.008% (w/w; 1:10,000 dilution when 50 mg plant material are extracted with 2 mL MeOH). LODs of analytes measured in positive ionization mode ranged from 0.3 to 0.5 ng/mL, translating to 0.012% and 0.02% (1:10,000 dilution), respectively. Biases and RSDs at the evaluated LOQs of 0.5 ng/mL, referring to 0.02% at the product level (1:10,000 dilution), lay within the acceptable range for all analytes. Linearity was shown with correlation factors (*R*^2^) of > 0.99 for calibrations of all analytes. The results for accuracy with precision and trueness met the defined criteria for all analytes at the investigated QC levels. All analytes met the defined criteria with maximum RSD_r_, RSD_(T)_, and bias of 16.8%, 16.0%, and − 19.3%, respectively. For detailed information on the validation results, see supplementary Table S3.

### Quantification of targeted cannabinoids

Mean contents (percentage; w/w) of the quantified cannabinoids for each variety are shown in Table 3. Detailed results including content ranges and corresponding standard deviations (SDs) can be found in the supplementary Table S4 (neutral and acid presented separately) and S5 (calculated total cannabinoid content, i.e., neutral + acid). Plant extractions were conducted in duplicate. The mean relative deviation of extracts of the same plant was ≤ 6.8% (median: 3.8%). RSD of the ISTDs was ≤ 2.3% throughout the presented analyses. When classifying into phenotypes I, II, and III [17], 14 varieties belonged to phenotype I (high-THC). The other four namely Pot of Gold nr. 11, Pot of Gold, GWS, and C7 × Thai additionally presented elevated CBD levels, therefore, belonging to phenotype II (intermediate type). CBDA (range: 0.03–9.5%), CBGA (range < LOQ–1.6%), CBCA (range: 0.11–0.26%), and THCVA (range: 0.03–1.7%) were detectable in all samples. CBDVA was only detectable in plants belonging to phenotype II. Several neutral cannabinoids were detected, but in considerably lower amounts than the corresponding acidic precursor. The neutral cannabinoid THC was detected at approximate levels ≤ 2.1% (range: 0.71–2.1%), CBG < 0.2% (range: 0.04–0.16%), and CBD < 1% (range: n.d.–0.6%), while the remaining cannabinoids (CBC, CBN, CBDV, THCV), if detected, were found at amounts < 0.1%. CBL and Δ8-THC (qualitatively screened) were not detected above their respective LODs in any sample. Inter-variety cannabinoid variability is assessable via the obtained SDs shown in Tables S2 and S3. The SDs of THC_Total_ (Table S3) ranged from ± 0.41% (Purple Punch, *n* = 3), showing the lowest variability, to ± 2.05% (Durban × Malawi, *n* = 8), presenting the highest SD. The highest difference between individual plants was observed for Amnesia × SFV (*n* = 2), with a mean THC_Total_ of 11.5% for plant 1 and 20.8% for plant 2.

**Table 3:** Mean quantitative results (two replicas per individual plant) expressed as content (%, w/w) for the investigated varieties. Columns are ordered from highest to lowest overall observed content; varieties are ordered from highest to lowest THCA content. CBL and ∆8-THC (qualitatively screened) were not detected above respective LODs in any sample and are therefore not shown. “n.d.” stands for “not detected” (< LOD)

Variety (n^a^)	THCA	CBDA	THC	CBD	CBGA	CBCA	THCVA	CBNA	CBG	CBC	CBN	CBDVA	THCV	CBDV
Amnesia S5 (3)	19.6	0.04	1.38	n.d	1.62	0.26	0.08	0.20	0.16	0.04	0.03	n.d	n.d	n.d
Lebi 2 (3)	19.3	0.04	1.15	n.d	0.42	0.24	0.07	0.19	0.15	0.02	0.03	n.d	n.d	n.d
Amnesia × SFV (2)	17.4	0.26	0.87	0.03	0.70	0.17	0.10	0.17	0.12	0.02	< LOQ	n.d	n.d	n.d
Purple Punch (3)	16.8	0.04	0.59	n.d	0.14	0.22	0.06	0.18	0.04	0.01	< LOQ	n.d	n.d	n.d
C7 (1)	15.6	0.05	0.93	n.d	0.20	0.13	0.10	0.23	0.08	0.01	0.04	n.d	n.d	n.d
Big Bud (3)	15.5	0.03	0.67	n.d	0.26	0.13	0.07	0.20	0.12	n.d	< LOQ	n.d	n.d	n.d
Amnesia (1)	15.5	0.03	0.93	n.d	0.62	0.14	0.05	0.16	0.08	0.02	0.03	n.d	n.d	n.d
Durban × Malawi (8)	15.4	0.05	1.04	< LOQ	0.18	0.13	0.17	0.14	0.05	< LOQ	< LOQ	n.d	< LOQ	n.d
Rascal OG (3)	13.4	0.03	1.94	n.d	0.10	0.19	0.04	0.17	0.04	0.03	0.06	n.d	n.d	n.d
Bubba Kush (3)	11.9	0.04	0.94	n.d	0.09	0.17	0.05	0.08	0.08	0.02	0.02	n.d	n.d	n.d
Malawi × Super Skunk (1)	11.5	0.03	1.46	n.d	1.07	0.43	1.73	0.11	0.26	0.03	0.03	n.d	0.24	n.d
Wappa (3)	11.3	0.03	1.85	n.d	0.22	0.18	0.26	0.16	0.05	0.04	0.06	n.d	0.05	n.d
SFV OG (3)	9.67	< LOQ	2.10	n.d	0.23	0.11	0.03	0.10	0.05	0.02	0.04	n.d	n.d	n.d
OG Kush (3)	8.68	< LOQ	0.65	n.d	< LOQ	0.17	< LOQ	0.08	0.04	< LOQ	< LOQ	n.d	n.d	n.d
Pot of Gold nr. 11 (2)	7.78	9.29	1.06	0.61	0.31	0.57	0.47	0.10	0.05	0.04	0.03	0.40	0.07	0.02
Pot of Gold (1)	7.54	9.48	1.26	0.72	0.37	0.57	0.47	0.09	0.06	0.05	0.03	0.42	0.08	< LOQ
GWS (1)	2.90	5.54	0.73	0.55	0.07	0.33	0.03	0.07	0.06	0.03	0.03	0.03	n.d	n.d
C7 × Thai (1)	2.88	6.12	0.71	0.60	0.05	0.42	0.03	0.06	0.05	0.04	0.03	0.04	n.d	n.d

^a^*n* total number of individual plants analyzed and available per variety

### Identification of untargeted additional compounds

The untargeted workflow detected 19 additional compounds. Including the 7 acidic cannabinoids, initially targeted in the negative ionization mode, a total of 26 compounds were detected. Table 4 shows all identified compounds, including theoretical molecular weights, measured [M-H]^−^ and mass errors as well as the herein detected MS^2^ fragments compared to MS^2^ fragments found in literature. Based on exact mass and matching MS^2^ spectra, nine compounds could be assigned to previously reported cannabinoids described in the literature [24, 46]. These compounds are therefore assigned with high confidence. For full verification of these results, however, analytical reference standards are required. Additional detected cannabinoids belonging to the THC family were two homologues of THCA, presenting different alkyl side chain lengths (THCA-C_1_, THCA-C_4_; also referred to as tetrahydrocannabutol abbreviated THCBA). Low signal intensities of these aforementioned compounds resulted in the detection of only one fragment each. This renders the annotation for THCA-C_1_ and THCA-C_4_ with higher uncertainty than for the other compounds that produced more characteristic MS^2^ spectra. THCA monomethyl ether (THCMA) was also detected. Cannabichromevarinic acid (CBCVA) and cannabigerovarinic acid (CBGVA) were identified as well. Additionally, two chromatographically separated isomers of the cannabinoid 6,7-epoxy-CBGA were found, as well as cannabigerolic acid monomethyl ether (CBGMA). Finally, cannabitriolic acid (CBTA) was identified. For 10 compounds (from here on termed *unknown 1* to *10*), the conclusive assignment was not possible due to missing MS^2^ spectra, resulting from low signal intensities and/or the lack of a matching known compound in literature. *Unknown 3* and *unknown 7*, both presenting a parent ion at [M-H]^−^  = 373.202 (*m/z*), match the signal of cannabielsoic acid (CBEA) as well as of other compounds reported by Berman et al. [24] Unfortunately, no MS^2^ spectra could be obtained for *unknown 3* and *unknown 7,* making a more conclusive assignment impossible. *Unknown 5* and *unknown 8* had the same elemental composition as the major cannabinoids THCA and CBDA, therefore, likely belonging to the cannabinoid class. A similar compound matching *unknown 5* and *unknown 8* was again reported by a study from Berman et al. [24]. Montone et al. [47] detected various isomers of cannabinoids applying an untargeted analysis. Accordingly, *unknown 8* that generated the same fragment at *m/z* 313 as THCA could be an isomer of THCA. *Unknown 10* with parent ion [M-H]^−^ at 325.145 (*m/z*) and proposed chemical formula of C_20_H_22_O_4_ matched the one expected for cannabivarinic acid (CBNVA). Structures of the herein tentatively detected cannabinoids are shown in supplementary material Table S2.

**Table 4:** List of all detected compounds using the untargeted data analysis. The herein detected fragments and (where indicated) published fragments are given in decreasing signal abundancies (excluding signals belonging to the unfragmented parent ion)

Compound	Formula	Theoretical [M-H]^−^ [*m/z*]	Measured [M-H]^−^ [*m/z*]	Mass error [ppm]	RT [min]	Detected fragments [*m/z*]	Published fragments [*m/z*]
6–7-Epoxy-CBGA (isomer 1)^b^	C_22_H_32_O_5_	375.2177	375.2179	0.4	1.02	357.21, 222.09, 273.19, 178.10	273, 357, 222, 313, 179 [46]
6–7-Epoxy-CBGA (isomer 2)^b^	C_22_H_32_O_5_	375.2177	375.2180	0.8	0.85	357.21^c^	273, 357, 222, 313, 179 [46]
CBCA^a^	C_22_H_30_O_4_	357.2071	357.2074	0.7	9.00	191.11, 313.22, 339.20, 179.11	191, 313, 339 [24]
CBCVA^b^	C_20_H_26_O_4_	329.1758	329.1758	-0.3	6.84	185.01, 285.19, 311.16	163, 311, 285, 151 [24]
CBDA^a^	C_22_H_30_O_4_	357.2071	357.2073	0.5	3.39	245.15, 339.20, 179.11, 311.20	245, 339, 311, 179 [24]
CBDVA^a^	C_20_H_26_O_4_	329.1758	329.1760	0.4	1.78	217.12, 311.17, 151.08, 283.17	217, 311, 151, 283 [24]
CBGA^a^	C_22_H_32_O_4_	359.2228	359.2229	0.3	4.31	341.21, 315.23, 297.22, 191.11	341, 315, 191, 297 [24]
CBGMA^b^	C_23_H_34_O_4_	373.2384	373.2387	0.7	9.07	329.25, 191.11, 245.15	355, 374, 329, 205 [24]
CBGVA^b^	C_20_H_28_O_4_	331.1915	331.1915	0.1	2.50	164.93, 313.18, 287.20, 217.19	313, 287, 164 [25]
CBNA^a^	C_22_H_26_O_4_	353.1758	353.1760	0.4	7.77	309.19, 279.14, 171.08	309, 279, 171 [25]
CBTA^b^	C_22_H_30_O_6_	389.1970	389.1969	-0.2	1.18	191.11, 327.21	309, 327, 285, 191 [25]
THCA^a^	C_22_H_30_O_4_	357.2071	357.2072	0.2	8.77	313.22, 245.15, 179.11	313, 339, 245 [25]
THCA-C_1_^c^	C_18_H_22_O_4_	301.1445	301.1446	0.2	3.40	257.16	257, 283, 189 [25]
THCA-C_4_^c^	C_21_H_28_O_4_	343.1915	343.1914	-0.4	7.47	257.16	299, 325, 231, 177 [25]
THCMA^b^	C_23_H_32_O_4_	371.2228	371.2229	0.3	8.48	259.17, 311.17, 193.12, 327.23	327, 371, 259, 205, 193 [25]
THCVA^a^	C_20_H_26_O_4_	329.1758	329.1758	-0.1	5.76	285.19, 217.12, 163.08	285, 311, 217, 163 [25]
Unknown 1	C_26_H_38_O_3_	397.2748	397.2745	-0.7	9.32	No MS^2^ obtained	n.a
Unknown 2	C_20_H_18_O_5_	337.1081	337.1083	0.4	0.66	No MS^2^ obtained	n.a
Unknown 3	C_22_H_30_O_5_	373.2020	373.2021	0.2	2.81	No MS^2^ obtained	n.a
Unknown 4	C_22_H_28_O_4_	355.1915	355.1917	0.6	8.64	No MS^2^ obtained	n.a
Unknown 5	C_22_H_30_O_4_	357.2071	357.2073	0.5	1.37	No MS^2^ obtained	n.a
Unknown 6	C_21_H_28_O_3_	327.1966	327.1964	-0.8	5.23	No MS^2^ obtained	n.a
Unknown 7	C_22_H_30_O_5_	373.2020	373.2020	0	3.14	No MS^2^ obtained	n.a
Unknown 8	C_22_H_30_O_4_	357.2071	357.2073	0.7	8.50	313.22, 215.45, 357.21	n.a
Unknown 9	C_22_H_32_O_6_	391.2126	391.2127	0.1	1.30	No MS^2^ obtained	n.a
Unknown 10	C_20_H_22_O_4_	325.1445	325.1450	1.6	4.50	No MS^2^ obtained	n.a

^a^Verified via comparison to certified reference material. ^b^Annotated via comparison to published [M-H]^−^ and MS^2^ spectra. ^c^Low signal intensity resulted in the detection of only one fragment that was distinguishable from noise, resulting in higher uncertainty in annotation. “n.a.” stands for not applicable

### Comparison of varieties – PCA resulting from the targeted and untargeted analysis

**Fig. 1:**
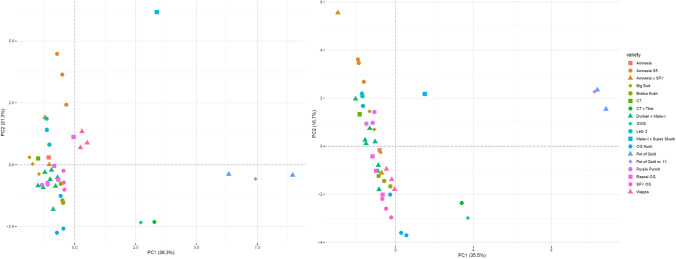
Scatter plots for the targeted analytes (left) and for the untargeted approach (right). Varieties presenting similar chemical fingerprints are clustering together, while distinct varieties are plotted further apart. Varieties belonging to phenotype II (Pot of Gold nr. 11, Pot of Gold, GWS, and C7 × Thai) are clearly distinguished from varieties of phenotype I. Slight differences between the resulting clusters are seen between the targeted (left) and untargeted (right) approach. Chemical subgroups are observable in both plots

**Fig. 2:**
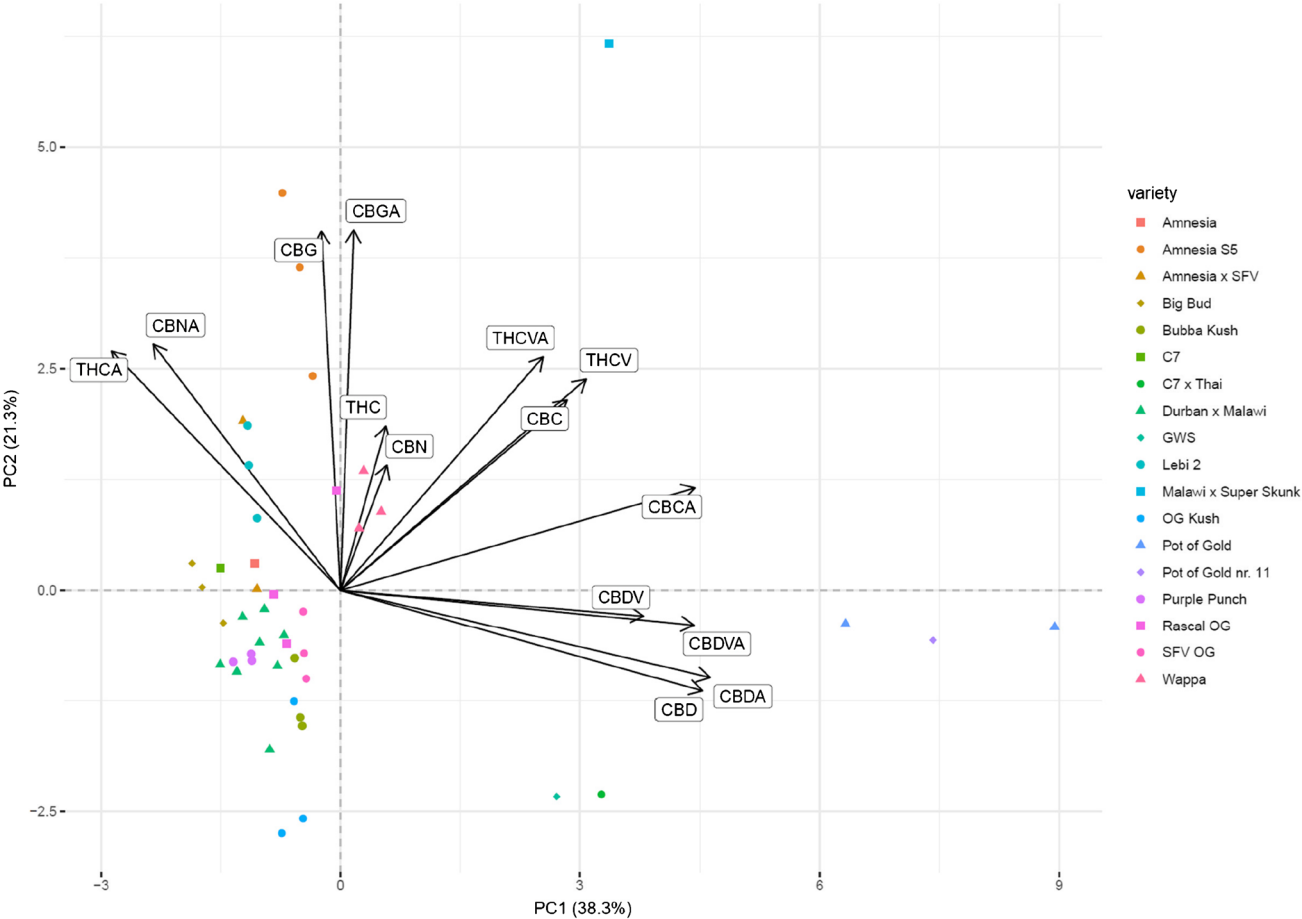
Loading plot (biplot) for the PCA of the targeted workflow. Eigenvectors are indicated by black arrows. The length and direction of these eigenvectors correspond to their contribution to the dimensions PC1 and PC2. Cannabinoids of the CBD type are largely contributing to the distinction between phenotypes I and II. THCA and CBNA are pointing in the opposite direction and are therefore more indicative for varieties belonging to phenotype I. CBG and CBGA are expressed at elevated levels for the variety Amnesia S5

**Fig. 3:**
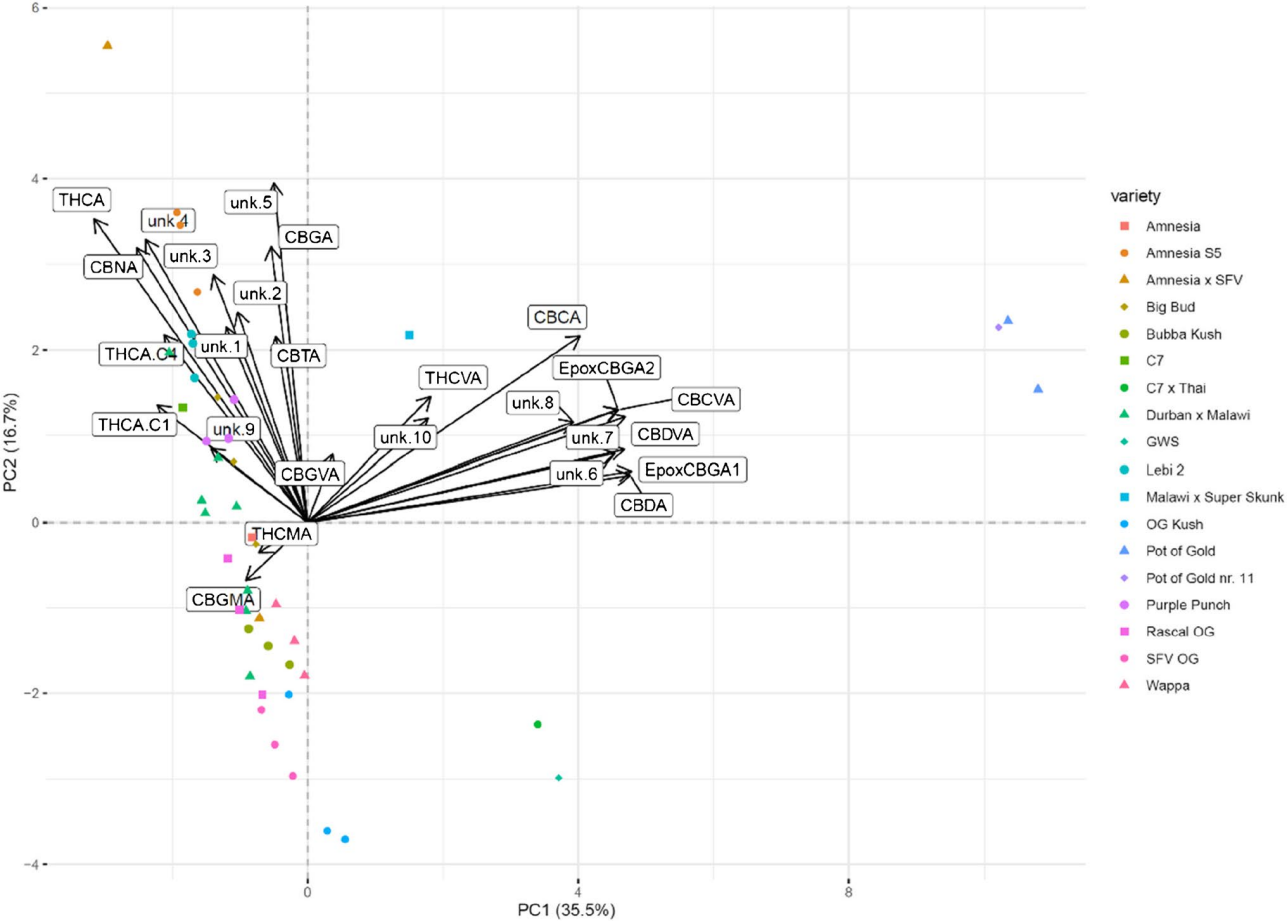
Loading plot (biplot) for the PCA of the targeted workflow. Eigenvectors are indicated by black arrows. The length and direction of these eigenvectors correspond to their contribution to the dimensions PC1 and PC2. Besides cannabinoids of the CBD family, 6,7-epoxy-CBGA isomers 1 and 2 were found to be highly indicative of the varieties Pot of Gold and Pot of Gold nr. 11. Note: “unkw.” stands for “unknown compound,” EpoxyCBGA1 = 6,7-epoxy-GBGA isomer 1, EpoxyCBGA1 = 6,7-epoxy-GBGA isomer 2

The obtained PCA scatter plots are presented in Fig. 1. The loading plot for the targeted analysis is presented in Fig. 2. The loading plot for the untargeted analysis is shown in Fig. 3. For the targeted approach, PC1 is contributing to 38.3% of variance and PC2 to 21.3%. Based on PCA of the data from the targeted workflow, the varieties belonging to the phenotype II, namely Pot of Gold, Pot of Gold nr. 11, GWS, and C7 × Thai, group in the first and fourth quadrants (counted from top right counterclockwise) of the scatter plot. The loading plot shows that the cannabinoids from the CBD family (CBD, CBDA, CBDV, CBDVA) are mostly contributing to the grouping of these varieties. CBCA is an additional eigenvector showing in this direction, meaning that CBCA was detected at higher levels in plants of phenotype II. In contrast, the eigenvectors for THCA and CBNA are pointing in the opposite direction of the ones of the CBD-type cannabinoids, indicating that these analytes behave counter-directional for these varieties. GWS and C7 × Thai presented similar chemical fingerprints, thus clustering in a distinct subgroup on the bottom right (fourth quadrant), attributable to their low contents of THCA. Cannabis varieties high in THC and, thus, belonging to phenotype I form one large cluster, which, apart from Wappa and Malawi × Super Skunk, are found in the second and third quadrant of the plot. Malawi × Super Skunk, not clustering with other varieties, expresses a unique chemical fingerprint compared to the other varieties. This is largely explained by its elevated THCVA levels, as seen in the loading plot. Nevertheless, additional subgroups within the large phenotype I cluster can be distinguished. For instance, Amnesia S5 and OG Kush are found on each end (top and bottom) of the cluster belonging to two different quadrants (second and third), thus, implying considerable differences in their chemical fingerprints largely attributable to their differences in their overall cannabinoid content, with Amnesia S5 presenting higher cannabinoid levels than OG Kush, e.g., THCA, THCVA, CBGA, CBG, and CBNA. Elevated CBGA and CBG levels are indicative for the variety Amnesia S5, as seen in the loading plot. Due to the small sample size of individual plants per variety (1–3 plants), calculation of the 95% confidence interval (95% CI) was only possible for the variety Durban × Malawi (*n* = 8). PCA scatter plots showing the 95% CI of Durban × Malawi are shown in supplementary Figs. S6 and S7.

PCA of the untargeted workflow showed similar results to the ones obtained with the targeted approach. The percentage of variance explained by PC1 and PC2 is slightly reduced to 35.5% and 16.7%, respectively. This can be explained by the introduction of a higher number of observations (i.e., compounds) with mixed discriminative value (reflected by compounds expressing short eigenvectors), which ultimately rendered the explanation of the variance of the whole dataset more difficult. Using the untargeted dataset, some varieties, for instance, Pot of Gold, Lebi 2, and OG Kush, clustered closer together than in the PCA plot generated from the targeted dataset. Thus, these varieties are better discriminated using the untargeted dataset. Others, however, are losing similarity when using the untargeted dataset, as seen for Amnesia S5 and Durban × Malawi. For these varieties, the additionally introduced compounds are showing higher variability than observed for the targeted analytes. Malawi × Super Skunk contained elevated levels of additional propyl-cannabinoids (C_3_) besides THCVA, namely CBGVA and *unknown 10* (likely to be CBNVA). Regarding the varieties Pot of Gold and Pot of Gold nr. 11 (a selection of Pot of Gold made by the producer with no given further information), as expected, very similar cannabinoid profiles were obtained. Upon investigation of the loading plot, the two 6,7-epoxy-CBGA isomers were shown to be indicative for these varieties. The relatively short eigenvectors belonging to CBGMA and THCMA point into a new direction (third quadrant), which has not been covered by any eigenvector for the targeted dataset. The variety Wappa did not express elevated CBGMA nor THCMA levels (Figure S9); thus, other low abundant or absent compounds of the newly introduced compounds with eigenvectors pointing to the top right has resulted in this variety being present in the untargeted approach in the third quadrant (while it was in the first quadrant for the targeted analysis). Additionally to Figs. 1, 2 and 3, heatmaps applying hierarchical clustering are shown in supplementary Figs. S8 and S9. These complementary multivariate analyses offer additional visualization of the data.

## Discussion

### Method validation

Despite the increasing demand for comprehensive product characterization [30], only a limited number of quantitative methods for the analysis of cannabis plant material spanning the range of 15 or more cannabinoids have been published so far [34, 48]. While ultraviolet (UV) or flame ionization detectors (FID) are commonly used for the robust quantification of major cannabinoids, the use of mass spectrometry has been suggested to improve specificity and widen the dynamic range [30, 48]. The latter is a prerequisite for the analysis of the lower abundant minor cannabinoids together with the typically high concentrated major cannabinoids [48].

For the presented study, deuterated and non-deuterated THC-metabolites 11-hydroxy-THC (OH-THC) and 11-nor-9-carboxy-THC (THC-COOH) were included as ISTDs for those cannabinoids where deuterated analogues were not commercially available. THCA and CBDA were calibrated using two separate calibration ranges, arising from the large concentration ranges required for these compounds. Although it would be most favorable to add the deuterated ISTDs before sample extraction, due to the required lower quantities and therefore reasonable costs, the addition of ISTDs at the final dilution step was chosen.

Sample dilution prior to analysis clearly influences the achievable LODs and LOQs. To fit analytes within a calibrated range, the injection of various dilutions poses an option; however, contamination of the analytical system and carry-over are limiting factors while, additionally, higher costs (resulting from material and longer runtimes) are disadvantageous. In the presented study, 14 out of the 15 validated analytes were detected, after applying a dilution of minimally 1:5000 to a sample of 50 mg plant material in 2 mL MeOH, which resulted in no contamination of the analytical system and no carry-over (assessed via blank injections between samples). The injection of higher concentrated samples was not possible due to the aforementioned limitations (contamination of the analytical system and carry-over). Selectivity was assessed via the measurement of diluted tea extracts and blank injections. The lack of a cannabinoid-free matrix hinders classical selectivity testing, which typically requires the measurement of blank matrices. Due to the same reason, preparation of matrix calibrators was not possible, requiring calibrators to be prepared in the solvent [48].

### Cannabinoid quantification

For the varieties belonging to phenotype I, the mean THC_Total_ content ranged from 10.6 to 18.5%. The United Nations Office on Drugs and Crime (UNODC) reported increasing THC_Total_ contents over the past decades in cannabis herbal preparations, with mean THC contents of approximately 10% in Europe and 15% in the USA in 2019 [21]. Thus, the herein investigated varieties belonging to phenotype I can be considered to span the range from average to high potency cannabis. Four varieties belonged to phenotype II, which is not believed to be commonly found on the recreational drug market [49]. Nonetheless, the varieties Pot of Gold and Pot of Gold nr. 11 both produced nearly equaling THC_Total_ and CBD_Total_ contents, therefore exhibited a similar THC/CBD profile as the marketed medicinal preparation Sativex® [34]. CBD itself is being investigated for various implications. It has been shown that CBD modulates the effects of THC; however, the interplaying effects of THC and CBD are not entirely understood [50]. In this study, CBL was not detectable in any samples. CBL is produced from CBC under heating, e.g., during smoking [1]. Therefore, under suitable storage conditions (cool and dry), the CBL content is expected to be very low.

The herein analyzed plants were cultivated and stored under identical and standardized conditions, therefore, eliminating changes introduced via heat, radiation, and prolonged storage periods, all influences which are believed to alter cannabinoid composition, e.g., by decarboxylation of acidic cannabinoids [2]. The standardized cultivation and storage conditions enable the assessment of inter-variety differences. In the presented study, Durban × Malawi and Anmesia × SFV showed high variability in their cannabinoid contents (as also seen in the PCA plots). In contrast to the other varieties, Durban × Malawi and Amnesia × SFV were grown from seeds and not cultivated from cuttings. Therefore, a higher variability of plant constituents was expected [1].

In a recent study, Scheunemann et al. [34] examined potential markers to distinguish medicinal from recreational cannabis intake, based on the analysis of 27 seized cannabis samples (all belonging to phenotype I) and various medicinal preparations, including Sativex®. The aforementioned authors developed and validated an analytical method for the detection and quantification of 16 cannabinoids, expanding the herein presented method with the analyte CBLA. Similar quantitative results as obtained in this study were obtained.

### Untargeted workflow

The introduction of high-resolution mass spectrometry considerably changed the field of cannabinoid analytics, largely due to the new possibility of complementing targeted approaches with untargeted analyses [30]. The untargeted analysis applied herein resulted in the detection of 19 additional compounds. Of those additional compounds, 9 were assigned to cannabinoids described in literature. However, as of today, reference materials of many minor cannabinoids are not readily available, especially for the acidic precursors (e.g., CBEA, CBTA, CBGMA) [30]. For full substance identification, regarding these tentatively assigned compounds, reference standards becoming available in the future should be measured. In recent years, the discovery of cannabidibutol (CBDB, CBD-C_4_) [51], Δ9-tetrahydrocannabutol (THCB, THC-C_4_) [52], cannabidiphorol (CBDP, CBD-C_7_), and Δ9-tetrahydrocannabiphorol (THCP, THC-C_7_) [53] in cannabis inflorescences attracted a lot of attention in the scientific community [54]. THCP levels in cannabis inflorescences after heating-induced decarboxylation have recently been published by Bueno et al. [54], who reported THCP levels ranging from 0.0023 to 0.0136%. In the presented study, THCBA (referred to as THCA-C_4_ in the presented study) was detected in 17 out of 18 varieties. CBDBA, CBDPA, and THCPA remained undetected, probably due to LODs not being low enough. Nonetheless, various additional cannabinoids have been tentatively identified using the herein presented approach. In a recent study, Montone et al. [47] employed a similar workflow using the Compound Discoverer™ software. The aforementioned authors were able to identify 121 phytocannabinoids, highlighting the potential of untargeted analyses in phytocannabinoid characterization.

### Comparison of varieties

Traditional classification based on THC and CBD contents [6, 16, 17] allowed differentiation of the investigated cannabis varieties into phenotypes I and II. As previously observed in other studies [6, 18, 24, 25, 27, 37, 39, 55], comprehensive analytical methods combined with multivariate statistical analyses, e.g., PCA, enabled for further subgrouping of cannabis varieties. The presented data concerning the PCA complemented the traditionally applied classification into phenotypes I, II, and III. The targeted and untargeted approach inarguably displayed a more refined and detailed image of the cannabinoid fingerprint. However, PCA also confirmed the important role of THCA and CBDA in the distinction of varieties, as these eigenvectors presented the highest divergence in the presented loading plot for the targeted data (Fig. 2). Comparing the PCA results obtained from the targeted versus the untargeted approach, slight differences in the observed clusters were seen: clustering was enhanced for some varieties, while it decreased for others depending on the dataset used. The untargeted approach resulted in the additional detection of further compounds, whereas the targeted approach has undergone method validation resulting in higher confidence in the obtained results and offering quantitative information. Consequently, regarding the characterization of cannabis varieties, both approaches have their eligibility.

Selected compounds were shown to be rather specific for some varieties, making them interesting as potential distinguishing markers. For instance, the 6,7-epoxy-CBGA isomers 1 and 2 are markers for the varieties Pot of Gold and Pot of Gold nr. 11 belonging to the phenotype II. Interestingly, THCMA and CBGMA resulted in eigenvectors pointing in a new direction in the loading plot (Fig. 3); however, the short length of the eigenvectors implies little discriminative value overall. The shorter alkyl-chain homologues of THC (THC-C1, THC-C4) were additional markers, distinct for plants of the phenotypes I, which was expected due to the close relation to THCA. CBGA and CBG levels contributed largely to the distinction within varieties belonging to phenotype I. THCVA was highly indicative for the variety Malawi × Super Skunk, which presented a unique chemical fingerprint.

### Limitations

The presented study was limited by the small number of samples per variety. As a result, the 95% CI for the PCA could only be calculated for one variety. The analytical procedure (e.g., chromatography and mass range of 200–400 m*/z*) was developed and optimized for cannabinoids. Other compound families (terpenoids and flavonoids) and other plant metabolites were, therefore, not the subject of this study.

While the standardized cultivation and storage conditions are regarded as an advantage in order to detect inter-variety differences, they might not be representative for the (illicit) recreational cannabis market. Ultimately, this limits the transferability of the presented results to settings encountered in forensic chemistry, where storage times and conditions of seized samples are generally not accessible. Finally, although popular names, e.g., Amnesia or White Widow [18], are commonly used to describe varieties, lack of classification as well as crossbreeding (especially for plants grown from seeds) must be considered when comparing results. The comparability of similar varieties obtained from various sources was beyond the scope of this study but is required to prove whether the herein reported results are transferable or not.

## Conclusion

The increasing availability of cannabis and derived products are posing the need for comprehensive analytical methods. The presented workflow comprised the expansion of a targeted method used for the quantification of 15 cannabinoids with an untargeted approach, employing in silico assisted identification of additional compounds. Thereby, new possibilities arising from high-resolution mass spectrometry in the field of *cannabinomics* are highlighted. PCA revealed additional subgroups, indicating distinct chemical composition of some varieties. Selected compounds, e.g., THVA, THCA homologues, and 6,7-epoxy-CBGA isomers 1 and 2, showed the potential to be used as distinguishing markers. Controlled cultivation and storage conditions enabled the assessment of intra- and inter-variety variability between plants. Expansion of the presented methodologies for chemical characterization of other materials than cannabis inflorescences, such as extracts, is conceivable, although requiring further validation. The presented approach provides a comprehensive and versatile means for cannabinoid fingerprinting on the product level. In-depth knowledge at the product level is key for product standardization, considered fundamental to ensure reproducible effects in humans (e.g., medicinal products) and may result in improved bioanalytical data interpretation in the medico-legal field.

## Supplementary Information

Below is the link to the electronic supplementary material.

Supplementary file1 (PDF 1697 KB)

## References

[1] ElSohly MA, Radwan MM, Gul W, Chandra S, Galal A, Kinghorn AD, Falk H, Gibbons S, Ji K (2017). Phytochemistry of Cannabis sativa L. Phytocannabinoids: unraveling the complex chemistry and pharmacology of Cannabis sativa.

[2] Gülck T, Møller BL (2020). Phytocannabinoids: origins and biosynthesis. Trends Plant Sci.

[3] Carliner H, Brown QL, Sarvet AL, Hasin DS (2017). Cannabis use, attitudes, and legal status in the U.S.: a review. Prev Med.

[4] Shanahan M, Cyrenne P (2021). Cannabis policies in Canada: how will we know which is best?. Int J Drug Policy.

[5] Manthey J, Freeman TP, Kilian C, López-Pelayo H, Rehm J (2021). Public health monitoring of cannabis use in Europe: prevalence of use, cannabis potency, and treatment rates. Lancet Reg Health Eur.

[6] Vásquez-Ocmín PG, Marti G, Bonhomme M, Mathis F, Fournier S, Bertani S (2021). Cannabinoids vs. whole metabolome: relevance of cannabinomics in analyzing Cannabis varieties. Anal Chim Acta.

[7] Ladha KS, Ajrawat P, Yang Y, Clarke H (2020). Understanding the medical chemistry of the cannabis plant is critical to guiding real world clinical evidence. Molecules.

[8] United Nations. UN commission reclassifies cannabis, yet still considered harmful. https://news.un.org/en/story/2020/12/1079132. Accessed 5 Jan 2022.

[9] Gould J (2015). The cannabis crop. Nature.

[10] Russo EB (2017). Cannabidiol claims and misconceptions. Trends Pharmacol Sci.

[11] Izzo AA, Borrelli F, Capasso R, Di Marzo V, Mechoulam R (2009). Non-psychotropic plant cannabinoids: new therapeutic opportunities from an ancient herb. Trends Pharmacol Sci.

[12] Pisanti S, Malfitano AM, Ciaglia E, Lamberti A, Ranieri R, Cuomo G (2017). Cannabidiol: state of the art and new challenges for therapeutic applications. Pharmacol Ther.

[13] Crippa JA, Guimarães FS, Campos AC, Zuardi AW (2018). Translational investigation of the therapeutic potential of cannabidiol (CBD): toward a new age. Front Immunol.

[14] Freeman TP, Hindocha C, Green SF, Bloomfield MAP (2019). Medicinal use of cannabis based products and cannabinoids. BMJ.

[15] Hanuš LO, Meyer SM, Muñoz E, Taglialatela-Scafati O, Appendino G (2016). Phytocannabinoids: a unified critical inventory. Nat Prod Rep.

[16] Small E, Chandra S, Lata H, ElSohly MA (2017). Classification of Cannabis sativa L. in relation to agricultural, biotechnological, medical and recreational utilization. Cannabis sativa L - botany and biotechnology.

[17] Small E, Beckstead HD (1973). Cannabinoid phenotypes in Cannabis sativa. Nature.

[18] Hazekamp A, Fischedick JT (2012). Cannabis - from cultivar to chemovar. Drug Test Anal.

[19] Fischedick JT (2017). Identification of terpenoid chemotypes among high (-)-trans-Δ(9)- tetrahydrocannabinol-producing Cannabis sativa L. cultivars. Cannabis Cannabinoid Res.

[20] Fournier G, Richez-Dumanois C, Duvezin J, Mathieu JP, Paris M (1987). Identification of a new chemotype in Cannabis sativa: cannabigerol - dominant plants, biogenetic and agronomic prospects. Planta Med.

[21] United Nations Office on Drugs and Crime. World Drug Report 2021. Booklet 3 - Drug market trends: Opioids, Cannabis. https://www.unodc.org/res/wdr2021/field/WDR21_Booklet_3.pdf. Accessed 3 Jan 2022.

[22] Baron EP (2018). Medicinal properties of cannabinoids, terpenes, and flavonoids in cannabis, and benefits in migraine, headache, and pain: an update on current evidence and cannabis science. Headache.

[23] Andre CM, Hausman J-F, Guerriero G (2016). Cannabis sativa: the plant of the thousand and one molecules. Front Plant Sci.

[24] Berman P, Futoran K, Lewitus GM, Mukha D, Benami M, Shlomi T (2018). A new ESI-LC/MS approach for comprehensive metabolic profiling of phytocannabinoids in Cannabis. Sci Rep.

[25] Berman P, Sulimani L, Gelfand A, Amsalem K, Lewitus GM, Meiri D (2020). Cannabinoidomics – an analytical approach to understand the effect of medical cannabis treatment on the endocannabinoid metabolome. Talanta.

[26] Russo EB (2011). Taming THC: potential cannabis synergy and phytocannabinoid-terpenoid entourage effects. Br J Pharmacol.

[27] Cerrato A, Citti C, Cannazza G, Capriotti AL, Cavaliere C, Grassi G (2021). Phytocannabinomics: untargeted metabolomics as a tool for cannabis chemovar differentiation. Talanta.

[28] Namdar D, Mazuz M, Ion A, Koltai H (2018). Variation in the compositions of cannabinoid and terpenoids in Cannabis sativa derived from inflorescence position along the stem and extraction methods. Ind Crops Prod.

[29] Aliferis KA, Bernard-Perron D. Cannabinomics: application of metabolomics in Cannabis (Cannabis sativa L.) research and development. Front Plant Sci. 2020;11(554). 10.3389/fpls.2020.00554. 10.3389/fpls.2020.00554 PMC7225349 32457786

[30] Capriotti AL, Cannazza G, Catani M, Cavaliere C, Cavazzini A, Cerrato A (2021). Recent applications of mass spectrometry for the characterization of cannabis and hemp phytocannabinoids: from targeted to untargeted analysis. J Chromatogr A.

[31] Calvi L, Pentimalli D, Panseri S, Giupponi L, Gelmini F, Beretta G (2018). Comprehensive quality evaluation of medical Cannabis sativa L. inflorescence and macerated oils based on HS-SPME coupled to GC–MS and LC-HRMS (q-exactive orbitrap®) approach. J Pharm Biomed Anal.

[32] Karschner EL, Swortwood-Gates MJ, Huestis MA (2020). Identifying and quantifying cannabinoids in biological matrices in the medical and legal cannabis era. Clin Chem.

[33] Scheunemann A, Elsner K, Germerott T, Groppa S, Hess C, Miederer I (2021). Identification of potential distinguishing markers for the use of cannabis-based medicines or street cannabis in serum samples. Metabolites.

[34] Scheunemann A, Elsner K, Germerott T, Hess C, Zörntlein S, Röhrich J (2021). Extensive phytocannabinoid profiles of seized cannabis and cannabis-based medicines – identification of potential distinguishing markers. Forensic Sci Int.

[35] Kraemer M, Madea B, Hess C (2019). Detectability of various cannabinoids in plasma samples of cannabis users: Indicators of recent cannabis use?. Drug Test Anal.

[36] Levin FR, Mariani JJ, Brooks DJ, Xie S, Murray KA (2010). Δ9-Tetrahydrocannabivarin testing may not have the sensitivity to detect marijuana use among individuals ingesting dronabinol. Drug Alcohol Depend.

[37] Fischedick JT, Hazekamp A, Erkelens T, Choi YH, Verpoorte R (2010). Metabolic fingerprinting of Cannabis sativa L., cannabinoids and terpenoids for chemotaxonomic and drug standardization purposes. Phytochemistry.

[38] Elzinga S, Fischedick J, Podkolinski R, Raber J. Cannabinoids and terpenes as chemotaxonomic markers in cannabis. Nat Prod Chem Res. 2015;3(4). 10.4172/2329-6836.1000181.

[39] Hazekamp A, Tejkalová K, Papadimitriou S (2016). Cannabis: from cultivar to chemovar II—a metabolomics approach to cannabis classification. Cannabis Cannabinoid Res.

[40] Slosse A, Van Durme F, Samyn N, Mangelings D, Vander HY (2020). Evaluation of data preprocessings for the comparison of GC–MS chemical profiles of seized cannabis samples. Forensic Sci Int.

[41] Pinto RC, Sussulini A (2017). Chemometrics methods and strategies in metabolomics. Metabolomics: from fundamentals to clinical applications.

[42] Raharjo TJ, Verpoorte R (2004). Methods for the analysis of cannabinoids in biological materials: a review. Phytochem Anal.

[43] Hazekamp A, Peltenburg A, Verpoorte R, Giroud C (2005). Chromatographic and spectroscopic data of cannabinoids from Cannabis sativa L. J Liq Chromatogr Relat Technol.

[44] De Vijlder T, Valkenborg D, Lemière F, Romijn EP, Laukens K, Cuyckens F (2018). A tutorial in small molecule identification via electrospray ionization-mass spectrometry: the practical art of structural elucidation. Mass Spectrom Rev.

[45] Lê S, Josse J, Husson F (2008). FactoMineR: An R package for multivariate analysis. J Stat Softw.

[46] Piccolella S, Crescente G, Formato M, Pacifico S. A cup of hemp coffee by Moka Pot from Southern Italy: an UHPLC-HRMS investigation. Foods. 2020;9(8). 10.3390/foods9081123. 10.3390/foods9081123 PMC7466224 32824076

[47] Montone CM, Cerrato A, Botta B, Cannazza G, Capriotti AL, Cavaliere C (2020). Improved identification of phytocannabinoids using a dedicated structure-based workflow. Talanta.

[48] McRae G, Melanson JE (2020). Quantitative determination and validation of 17 cannabinoids in cannabis and hemp using liquid chromatography-tandem mass spectrometry. Anal Bioanal Chem.

[49] ElSohly MA, Mehmedic Z, Foster S, Gon C, Chandra S, Church JC (2016). Changes in cannabis potency over the last 2 decades (1995–2014): analysis of current data in the United States. Biol Psychiatry.

[50] Freeman AM, Petrilli K, Lees R, Hindocha C, Mokrysz C, Curran HV (2019). How does cannabidiol (CBD) influence the acute effects of delta-9-tetrahydrocannabinol (THC) in humans? A systematic review. Neurosci Biobehav Rev.

[51] Citti C, Linciano P, Forni F, Vandelli MA, Gigli G, Laganà A (2019). Analysis of impurities of cannabidiol from hemp. Isolation, characterization and synthesis of cannabidibutol, the novel cannabidiol butyl analog. J Pharm Biomed Anal.

[52] Linciano P, Citti C, Russo F, Tolomeo F, Laganà A, Capriotti AL (2020). Identification of a new cannabidiol n-hexyl homolog in a medicinal cannabis variety with an antinociceptive activity in mice: cannabidihexol. Sci Rep.

[53] Citti C, Linciano P, Russo F, Luongo L, Iannotta M, Maione S (2019). A novel phytocannabinoid isolated from Cannabis sativa L. with an in vivo cannabimimetic activity higher than Δ9-tetrahydrocannabinol: Δ9-tetrahydrocannabiphorol. Sci Rep..

[54] Bueno J, Greenbaum EA (2021). (−)-trans-Δ9-Tetrahydrocannabiphorol content of Cannabis sativa inflorescence from various chemotypes. J Nat Prod.

[55] Elzinga S, Fischedick J, Podkolinski R, Raber J. Cannabinoids and terpenes as chemotaxonomic markers in cannabis. Nat Prod Chem Res. 2015;3. 10.4172/2329-6836.1000181.

