# Supplementary material for: Beyond Δ9-tetrahydrocannabinol and cannabidiol: chemical differentiation of cannabis varieties applying targeted and untargeted analysis
Source: Anal Bioanal Chem. 2022 Apr 5;414(13):3847–62. doi: 10.1007/s00216-022-04026-2 (PMC9061671; doi:10.1007/s00216-022-04026-2)
Supplement: Supplementary file 1 — Supplementary file1 (PDF 1697 KB) [file 216_2022_4026_MOESM1_ESM.pdf]

Supporting information:

Beyond Δ9-tetrahydrocannabinol and cannabidiol: Chemical differentiation of cannabis varieties applying targeted and untargeted analysis

Authors: Manuela Carla Monti; Priska Frei; Sophie Weber; Eva Scheurer; Katja Mercer-Chalmers-Bender*

Affiliation: Institute of Forensic Medicine, Department of Biomedical Engineering, University of Basel, Basel, Switzerland

*Corresponding author, *katja.mercer-chalmers-bender@unibas.ch*

List of Supplementary Figures

Figure S1 Exemplary extracted ion chromatograms (p. 9)
Figure S2 Advanced parameters applied for the untargeted data processing in the Compound Discoverer™ Software (p. 10)
Figure S3 Source code for the principal component analysis (PCA) and plotting (loading plots) in R (p. 11)
Figure S4 Source code data normalization (z-score transformation) and hierarchical clustering in R (p. 12)
Figure S5 Exemplary mass trace of Δ9-THC and its isomer Δ8-THC (p. 12)
Figure S6 PCA loading plot of the targeted analysis showing 95% confidence interval (95%-CI) for Duban x Malawi (p. 16)
Figure S7 PCA loading plot of the untargeted analysis showing 95% confidence interval (95%-CI) for Duban x Malawi (p. 17)
Figure S8 Heatmap applying hierarchical clustering of the targeted dataset (contents) (p. 18)
Figure S9 Heatmap applying hierarchical clustering of the untargeted dataset (area) (p. 19)

List of Supplementary Tables

Table S1 List of certified reference material including vendors (p. 2)
Table S2 Structures of cannabinoids contained in the targeted method and detected during the untargeted workflow (p. 3 - 8)
Table S3 Summary of validation results (p. 13)
Table S4 Cannabinoid contents (acid and free shown separately) of the investigated cannabis varieties (p.14)
Table S5 Total cannabinoid contents (acid + free) of the investigated cannabis varieties (p. 15)

Table S1 List of the used certified reference material (CRM) and respective vendors

Analyte	Full name	Vendor
Δ9-THC (THC)	Δ^9-Tetrahydrocannabinol	Lipomed (Arlesheim, Switzerland)
Δ8-THC	Δ^8-Tetrahydrocannabinol	Lipomed (Arlesheim, Switzerland)
D_3-Δ9-THC	Deuterated THC	Lipomed (Arlesheim, Switzerland)
THCA	THC-acid	Lipomed (Arlesheim, Switzerland)
THCV	Tetrahyrocannabidivarin	Merck (Buchs, Switzerland)
THCVA	Tetrahyrocannabidivarin acid	Merck (Buchs, Switzerland)
CBD	Cannabidiol	Lipomed (Arlesheim, Switzerland)
D_3-CBD	Deuterated CBD	Lipomed (Arlesheim, Switzerland)
CBDA	CBD-acid	Lipomed (Arlesheim, Switzerland)
CBDV	Cannabidivarin	Merck (Buchs, Switzerland)
CBDVA	Cannabidivarinic acid	Merck (Buchs, Switzerland)
CBN	Cannabinol	Lipomed (Arlesheim, Switzerland)
D_3-CBN	Deuterated CBN	Lipomed (Arlesheim, Switzerland)
CBNA	CBN-acid	Merck (Buchs, Switzerland)
CBC	Cannabichromene	Lipomed (Arlesheim, Switzerland)
D_9-CBC	Deuterated CBC	Cayman Chemical Company (Michigan, USA)
CBCA	Cannabichromenic acid	Merck (Buchs, Switzerland)
CBG	Cannabigerol	Lipomed (Arlesheim, Switzerland)
CBGA	CBG-acid	Merck (Buchs, Switzerland)
CBL	Cannabicyclol	Merck (Buchs, Switzerland)
OH-THC	11-hydroxy-THC	Lipomed (Arlesheim, Switzerland)
OH-THC-D3	Deuterated 11-hydroxy-THC	Lipomed (Arlesheim, Switzerland)
THC-COOH	11-nor-9-carboxy-THC	Lipomed (Arlesheim, Switzerland)
THC-COOH-D9	Deuterated 11-nor-9-carboxy-THC	Lipomed (Arlesheim, Switzerland)

Table S2 Structures, CAS numbers (where available), and chemical formula of cannabinoids contained in the targeted method and detected during the untargeted workflow (tentatively identified). "n.a." stands for not applicable

**THC-type**			
**Analyte**	**CAS**	**Formula**	**Full name and chemical structure**
Δ9-THC	1972-08-3	$C_{21}H_{30}O_2$	**Δ^{9}-Tetrahydrocannabinol** 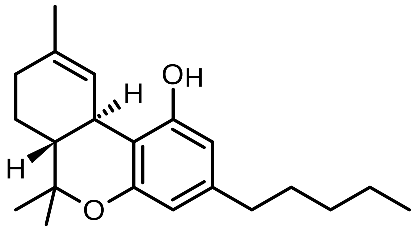
Δ8-THC	5957-75-5	$C_{21}H_{30}O_2$	**Δ^{8}-Tetrahydrocannabinol** 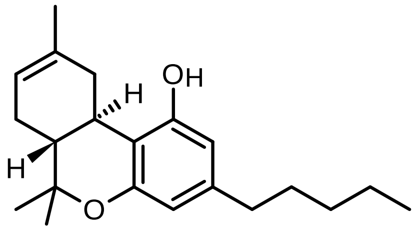
THCA	23978-85-0	$C_{22}H_{30}O_4$	**Δ^{9}-Tetrahydrocannabinolic acid** 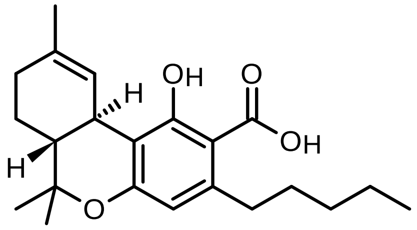
THCV	31262-37-0	$C_{19}H_{26}O_2$	**Tetrahyrocannabidivarin** 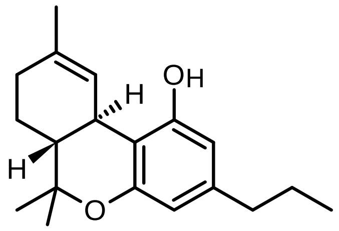
THCVA	39986-26-0	$C_{20}H_{26}O_4$	**Tetrahyrocannabidivarin acid** 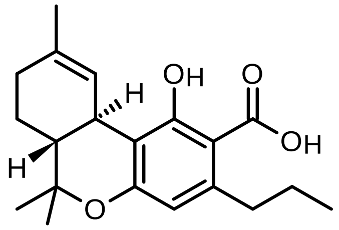

THCMA	n.a.	$C_{23}H_{32}O_4$	**Tetrahyrocannabinolic acid monomethyl ester**<br>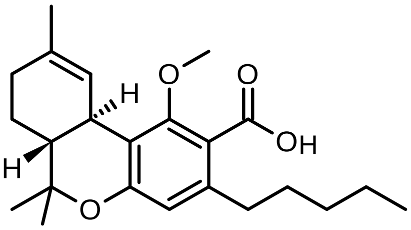
THCBA<br>THCA-C4	n.a.	$C_{21}H_{28}O_4$	**Tetrahydrocannabutolic acid**<br>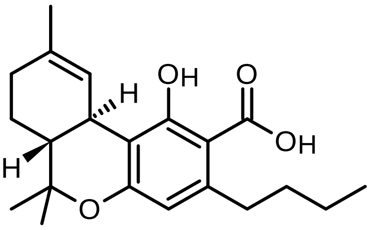
THCA-C1	n.a.	$C_{18}H_{22}O_4$	**Tetrahydrocannabinolic acid-C1**<br>**= Tetraydrocannabiorcolic acid**<br>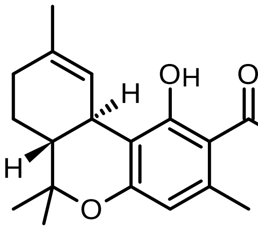

**CBD-type**			
**Analyte**	**CAS**	**formula**	**Full name and chemical structure**
CBD	13956-29-1	$C_{21}H_{30}O_2$	**Cannabidiol**<br>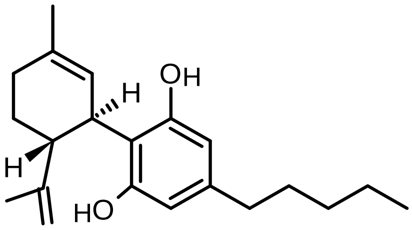
CBDA	1244-58-2	$C_{22}H_{30}O_4$	**Cannabidiolic acid**<br>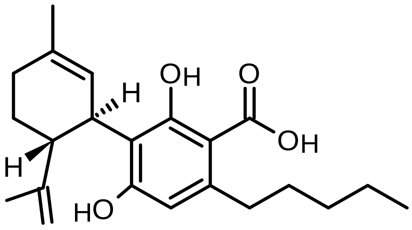

CBDV	24274-48-4	$C_{19}H_{26}O_2$	**Cannabidivarin** 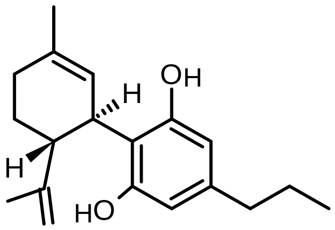
CBDVA	31932-13-5	$C_{20}H_{26}O_4$	**Cannabidivarinic acid** 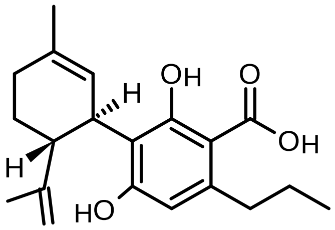

**CBG-type**			
**Analyte**	**CAS**		**Full name and chemical structure**
CBG	25654-31-3	$C_{21}H_{32}O_2$	**Cannabigerol** 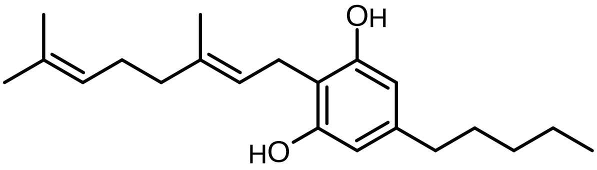
CBGA	25555-57-1	$C_{22}H_{32}O_4$	**Cannabigerolic acid** 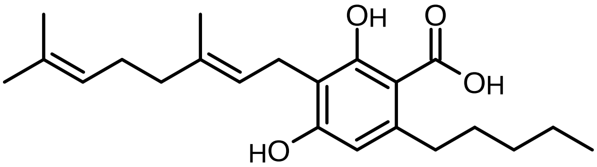
CBGVA	64924-07-8	$C_{20}H_{28}O_4$	**Cannabigerivarinic acid** 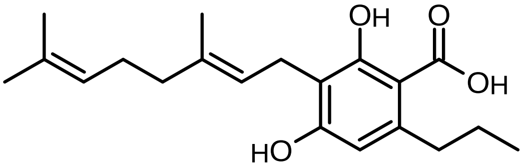

CBGMA	29624-08-6	$C_{23}H_{34}O_4$	**Cannabigerolic acid monomethyl ester** 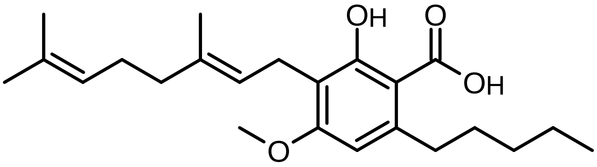
6,7-Epoxy-CBGA	n.a.	$C_{22}H_{32}O_5$	**6,7-Epoxy-cannabigerolic acid isomers 1 and 2** 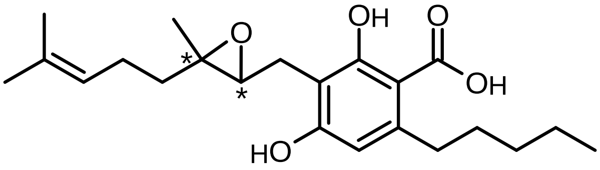

**CBN-type**			
**Analyte**	**CAS**		**Full name and chemical structure**
CBN	13956-29-1	$C_{21}H_{26}O_2$	**Cannabinol** 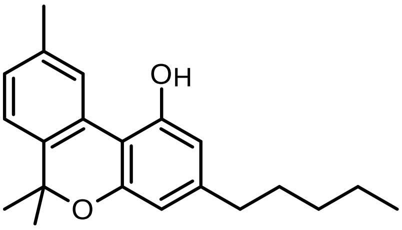
CBNA	2808-39-1	$C_{22}H_{26}O_4$	**Cannabinolic acid** 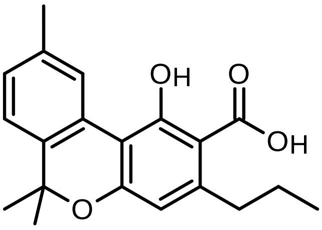
CBNVA	n.a.	$C_{20}H_{22}O_4$	**Cannabinovarinic acid** 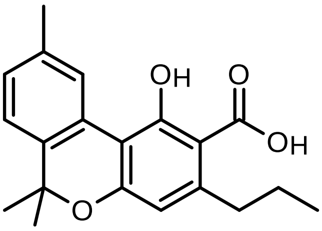

CBC-type			
**Analyte**	**CAS**		**Full name and chemical structure**
CBC	20675-51-8	$C_{21}H_{30}O_2$	**Cannabichromene** 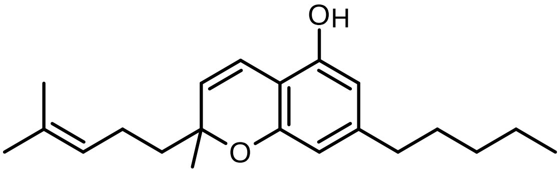
CBCA	185505-15-1	$C_{22}H_{30}O_4$	**Cannabichromenic acid** 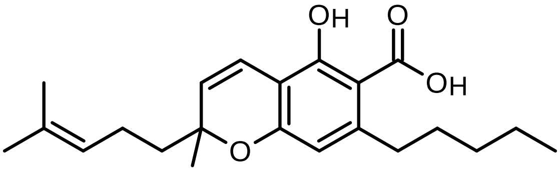
CBCVA	64898-02-8	$C_{20}H_{26}O_4$	**Cannabichromevarinic acid** 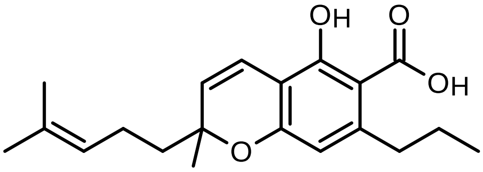

CBL-type			
**Analyte**	**CAS**	**Formula**	**Full name and chemical structure**
CBL	21366-63-2	$C_{21}H_{30}O_2$	**Cannabicyclol** 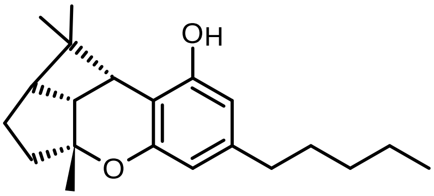
CBLA	n.a.	$C_{22}H_{30}O_4$	**Cannabicyclolic acid** 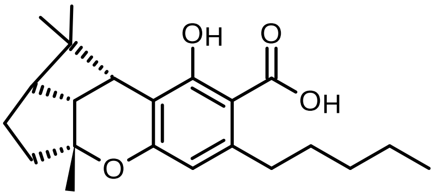

CBT-type			
**Analyte**	**CAS**	**Formula**	**Full name and chemical structure**
CBTA	n.a.	$C_{22}H_{30}O_6$	**Cannabitriolic acid**<br>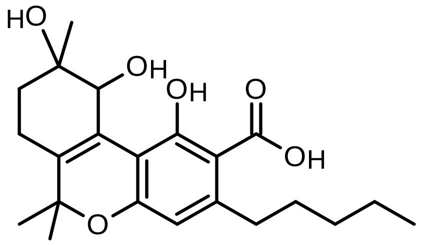

CBE-type			
**Analyte**	**CAS**	**Formula**	**Full name and chemical structure**
CBEA	n.a.	$C_{22}H_{30}O_5$	**Cannabielsoinic acid**<br>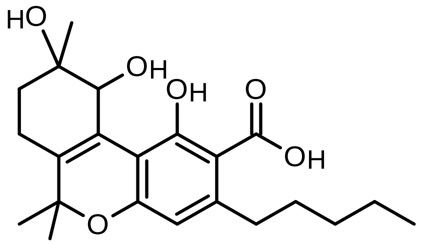

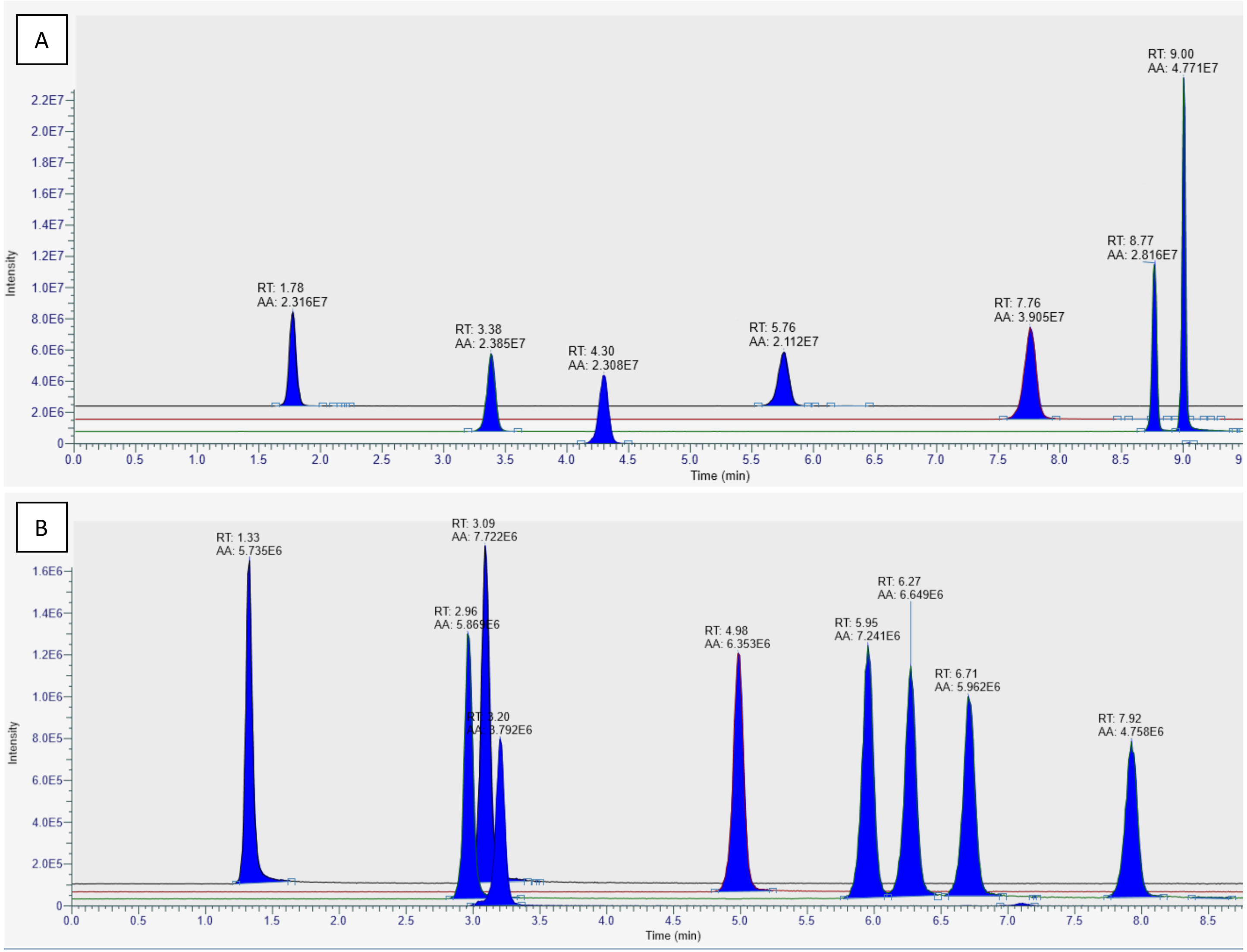

Figure S1 Exemplary extracted ion chromatograms (QC samples at 80 ng/mL including Δ8-THC at 80 ng/mL, 5 ppm mass tolerance). A: negative ionization mode; CBCVA (1.78 min), CBDA (3.38 min), CBGA (4.30 min), THCVA (5.76 min), CBNA (7.76 min), THCA (8.77 min), CBCA (9.05 min). B: positive ionization mode; CBDV (1.33 min), CBD (2.96 min), THCV (3.09 min), CBG (3.20 min), CBN (4.98), Δ9-THC (5.95 min), Δ8-THC (6.27 min), CBL (6.71 min), CBC (7.92 min)

Select Spectra:

**1. General Settings**	
Precursor Selectio	Use MS1 Precursor
Provide Profile Sp	Automatic
**2. Spectrum Properties Filter**	
Lower RT Limit	0
Upper RT Limit	0
First Scan	0
Last Scan	0
Ignore Specified S	
Lowest Charge Sta	0
Highest Charge St	0
Min. Precursor Ma	0 Da
Max. Precursor M	5000 Da
Total Intensity Thr	0
Minimum Peak Co	1
**3. Scan Event Filters**	
Mass Analyzer	(Not specified)
MS Order	Any
Activation Type	(Not specified)
Min. Collision Ene	0
Max. Collision Ene	1000
Scan Type	Any
Polarity Mode	(Not specified)
**4. Peak Filters**	
S/N Threshold (FT-	1.5
**5. Replacements for Unrecognized Properties**	
Unrecognized Cha	1
Unrecognized Ma	ITMS
Unrecognized MS	MS2
Unrecognized Acti	CID
Unrecognized Pol	-
Unrecognized MS	60000
Unrecognized MS	30000

Align Retention Times:

**1. General Settings**	
Alignment Model	Adaptive curve
Maximum Shift [m	2
Mass Tolerance	5 ppm

Create Mass Trace:

**1. General Settings**	
Trace Type	BPC
MS Order	MS1
Polarity	+
Custom Label	
**2. XIC Settings**	
Mass [Da]	0
Mass Tolerance	5 ppm

Detect Compounds:

**1. General Settings**	
Mass Tolerance [p	5 ppm
Intensity Toleranc	30
S/N Threshold	3
Min. Peak Intensit	10000
Ions	[M-H]-1
Min. Element Cou	C H
Max. Element Cou	C90 H190 Br3 Cl4 K2 N10 Na2

Group Compounds:

**1. Compound Consolidation**	
Mass Tolerance	5 ppm
RT Tolerance [min]	0.2
**2. Fragment Data Selection**	
Preferred Ions	[M-H]-1

Search Mass Lists:

**1. Search Settings**	
Mass Lists	\Cannabinoids neg.massList
Use Retention Tim	True
RT Tolerance [min]	0.5
Mass Tolerance	5 ppm

Mark Background Compounds:

**1. General Settings**	
Max. Sample/Blan	5
Max. Blank/Sampl	0
Hide Background	True

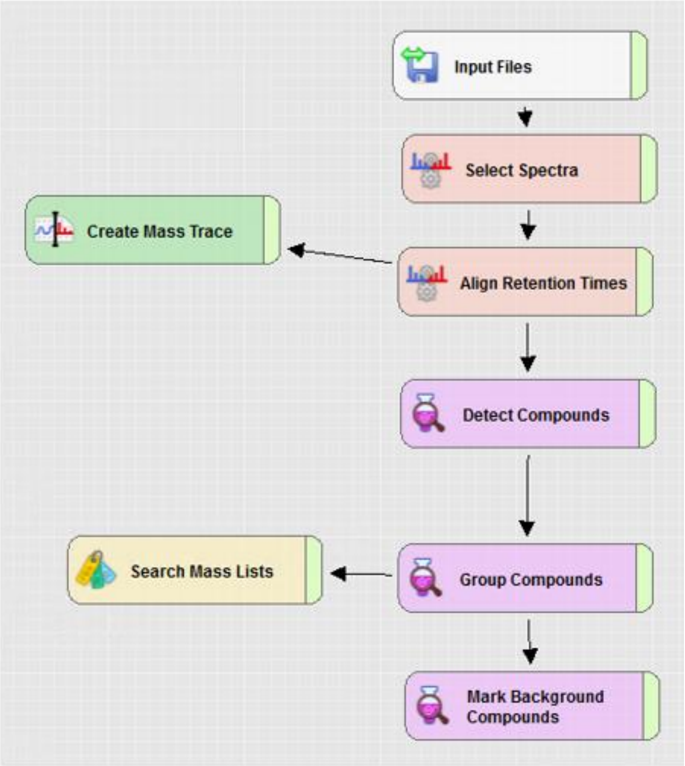

Figure S2 Advanced parameters applied for the untargeted data processing in the Compound Discoverer (Version 3.1.0.305) Software from Thermo Fisher Scientific (Reinach, Switzerland)

```
library(ggplot2)
library(FactoMineR)
library(ggrepel)

mydata.pca <- FactoMineR::PCA(mydata, graph = FALSE)
plotdata <- data.frame(mydata.pca$ind$coord)
plotdata$variety <- mydata$Variety
x_dim <- 1
y_dim <- 2
scale_factor <- 5.
loadings <- data.frame(mydata.pca$var$coord) * scale_factor
loadings$label <- row.names(loadings)
loadings$origin <- 0.
ggplot(plotdata, aes_string(x=paste0('Dim.',x_dim), y=paste0('Dim.', y_dim))) +
  geom_vline(xintercept = 0, linetype=2, alpha=0.2) +
  geom_hline(yintercept = 0, linetype=2, alpha=0.2) +
  geom_segment(data = loadings, aes_string(xend=paste0('Dim.',x_dim), yend=paste0('Dim.', y_dim),
x='origin', y='origin'), arrow = arrow(length = unit(10, units = 'pt'))) +
  geom_label_repel(data=loadings, aes_string(x=paste0('Dim.',x_dim), y=paste0('Dim.', y_dim),
label='label'), ) +
  geom_point(aes(col=variety, shape=variety), size=4) +
  theme_bw() +
  xlab(paste0('PC',x_dim,' (',round(mydata.pca$eig[x_dim,'percentage of variance'],1),'%)')) +
  ylab(paste0('PC',y_dim,' (',round(mydata.pca$eig[y_dim,'percentage of variance'],1),'%)')) +
  theme(panel.border = element_blank()) +
  scale_shape_manual(values = rep(c(15:19),length.out=length(unique(plotdata$variety))))
```

```
sessionInfo()
gplots_3.1.1    ggrepel_0.9.1    FactoMineR_2.4
```

Figure S3 Source code for the principal component analysis (PCA) and plotting (scatter plots, loading plots; biplots) in R (version 3.4.3)

```
library(ggplot2)
library(cluster)
library(ggrepel)
library(gplots)
library(RColorBrewer)

mydata_norm <- scale(mydata ,center = T,scale = T)
row.names(mydata_norm) <- mydata$Variety
heatmap.2(mydata_norm,  cexRow = 0.5, cellnote = mydata[,-15], notecex = 0.5, notecol = 'white', col
= colors, breaks = seq(-3,3,length.out=51), margins = c(5,8), trace = 'none')

________________________________________________________________________________
sessionInfo()
RColorBrewer_1.1-2      gplots_3.1.1      ggrepel_0.9.1    ggplot2_3.3.5      cluster_2.1.2
```

Figure S4 Source code for data normalization (z-score transformation) and hierarchical clustering in R (version 3.4.3)

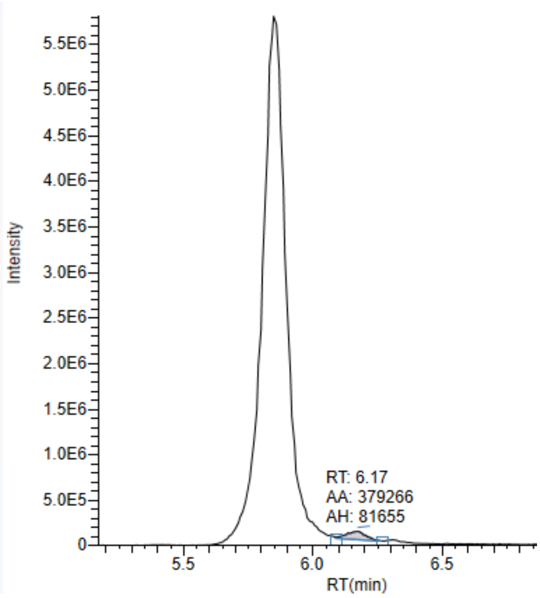

Figure S5 Mass trace (m/z 315.23157; 5 ppm mass error, positive ionization mode) corresponding to Δ9-THC at 500 ng/mL (5.85 min) and its isomer Δ8-THC at 5 ng/mL (6.17 min). Sample was produced from certified reference material

Table S3 Summary of validation results, including limit of detection (LOD), limit of quantification (LOQ), intra-day (RSD_r) and inter-day ($RSD_{(T)}$), bias, and linearity range. Target values: QC_{low1}= 0.8 ng/mL (0.032%, w/w); QC_{low2} = 3.0 ng/mL (0.12%, w/w)*, QC_{medium} = 80 ng/mL (3.2%, w/w)*; QC_{high} = 400 ng/mL (16.0%, w/w)*; *considering a 50 mg sample extracted with 2 mL MeOH and 1 : 10,000 dilution of the sample)*

Analyte	LOD [ng/mL]	LOQ [ng/mL]	QC level	RSD_r [%]	$RSD_{(T)}$ [%]	Bias [%]
CBC	0.5	0.5	low_1	16.8	16.0	-12.4
			low_2	2.9	7.2	4.3
			medium	1.2	1.3	7.9
CBCA	0.2	0.5	low_1	4.1	8.8	-19.3
			low_2	5.7	7.5	-12.0
			medium	2.7	6.0	-7.2
CBD	0.3	0.5	low_1	4.1	5.0	-2.5
			low_2	2.9	5.0	0.8
			medium	0.6	0.9	6.6
CBDA	0.2	0.5	low_1	2.4	6.3	-12.0
			low_2	3.6	3.6	-0.4
			medium	3.4	6.6	1.0
			high	1.2	2.9	-4.9
CBDV	0.3	0.5	low_1	4.7	12.9	-4.7
			low_2	2.7	6.5	1.5
			medium	1.2	1.2	0.8
CBDVA	0.2	0.5	low_1	2.9	5.4	-9.6
			low_2	4.0	4.6	-1.7
			medium	3.0	6.6	-0.5
CBG	0.4	0.5	low_1	7.0	13.9	-13.5
			low_2	2.7	3.4	2.9
			high	2.3	2.3	1.3
CBGA	0.2	0.5	low_1	2.1	5.1	-9.7
			low_2	4.3	4.3	1.0
			medium	2.3	5.4	3.0
CBL	0.4	0.5	low_1	6.8	12.6	-10.1
			low_2	2.7	5.9	-3.6
			medium	0.8	1.0	-0.2
CBN	0.4	0.5	low_1	4.6	12.3	-8.6
			low_2	2.9	4.1	-0.9
			medium	1.1	1.1	2.3
CBNA	0.2	0.5	low_1	1.4	7.6	-7.4
			low_2	8.9	8.9	-4.7
			medium	3.5	5	<0.1
THC	0.3	0.5	low_1	4.4	9.4	-4.3
			low_2	4.6	5.1	2.0
			medium	0.5	1.0	2.7
THCA	0.2	0.5	low_1	4.1	12.0	-17.9
			low_2	4.8	5.1	1.2
			medium	2.7	3.5	3.9
			high	1.7	3.5	-4.8
THCV	0.3	0.5	low_1	6.1	7.9	-4.1
			low_2	3.6	5.8	3.7
			medium	0.7	1.5	3.2
THCVA	0.2	0.5	low_1	2.8	6.2	-8.4
			low_2	5.2	5.4	-8.6
			medium	2.4	5.2	-3.7

Table S4 Cannabinoid contents of different cannabis varieties. CBL and Δ8-THC were not detected in any of the samples. Standard deviation and range (shown in brackets) are only given, if number of replicas (n) ≥ 3. Limit of quantification (LOQ) of all analytes was 0.02% (considering a 50 mg sample extracted with 2 mL MeOH and 1 : 10,000 dilution of the sample)

		**Mean ± standard deviation (range) [%]**													
**Variety**	**n**	**CBC**	**CBCA**	**CBD**	**CBDA**	**CBDV**	**CBDVA**	**CBG**	**CBGA**	**CBN**	**CBNA**	**Δ⁹-THC**	**Δ⁹-THCA**	**THCV**	**THCVA**
Amnesia	1	<LOQ	0.14	n.d.	0.03	n.d.	n.d.	0.08	0.62	0.03	0.16	0.93	15.50	n.d.	0.05
Amnesia S5	3	0.05 ± 0.01<br>(0.04-0.05)	0.26 ± 0.02<br>(0.24-0.28)	n.d.	0.04 ± 0.01<br>(0.04-0.05)	n.d.	n.d.	0.16 ± 0.04<br>(0.12-0.20)	1.62 ± 0.20<br>(1.34-1.80)	0.03 ± 0.01<br>(0.03-0.04)	0.20 ± 0.02<br>(0.17-0.20)	1.38 ± 0.19<br>(1.16-1.64)	19.6 ± 1.77<br>(17.1-20.9)	n.d.	0.08 ± 0.01<br>(0.08-0.09)
Amnesia x SFV	2	<LOQ, <LOQ	0.06-0.28	n.d., 0.05	0.04, 0.47	n.d., n.d.	n.d., n.d.	0.09, 0.16	0.68, 0.73	<LOQ, 0.03	0.15, 0.20	0.74, 1.00	12.3, 22.6	n.d., n.d.	0.07, 0.13
Big Bud	3	n.d.	0.13 ± 0.01<br>(0.12-0.14)	n.d.	0.03 ± 0.00<br>(0.03-0.04)	n.d.	n.d.	0.12 ± 0.01<br>(0.12-0.14)	0.26 ± 0.03<br>(0.22-0.28)	detected[a]<br>(<LOQ-0.02)	0.20 ± 0.02<br>(0.17-0.21)	0.67 ± 0.04<br>(0.63-0.73)	15.5 ± 1.29<br>(14.0-17.2)	n.d.	0.07 ± 0.01<br>(0.06-0.07)
Bubba Kush	3	detected[b]<br>(<LOQ-0.02)	0.17 ± 0.02<br>(0.15-0.19)	n.d.	0.04 ± 0.02<br>(0.02-0.06)	n.d.	n.d.	0.08 ± 0.01<br>(0.07-0.09)	0.09 ± 0.01<br>(0.07-0.09)	detected[c]<br>(<LOQ-0.02)	0.08 ± 0.01<br>(0.07-0.09)	0.94 ± 0.14<br>(0.76-1.09)	11.9 ± 1.46<br>(10.5-13.9)	n.d.	0.05 ± 0.00<br>(0.04-0.05)
C7	1	<LOQ	0.13	n.d.	0.05	n.d.	n.d.	0.08	0.20	0.04	0.23	0.93	15.5	n.d.	0.10
C7 x Thai	1	0.04	0.42	0.60	6.12	n.d.	0.04	0.05	0.05	0.03	0.06	0.71	2.88	n.d.	0.03
Durban x Malawi	8	detected[d]<br>(n.d.-<LOQ)	0.13 ± 0.01<br>(0.11-0.15)	detected[e]<br>(n.d.-<LOQ)	0.05 ± 0.02<br>(0.03-0.10)	n.d.	n.d.	0.05 ± 0.01<br>(0.03-0.06)	0.18 ± 0.06<br>(0.09-0.27)	detected[f]<br>(<LOQ-0.03)	0.14 ± 0.02<br>(0.09-0.16)	1.04 ± 0.35<br>(0.59-1.21)	15.4 ± 2.51<br>(11.2-18.8)	detected[g]<br>(n.d.-0.03)	0.17 ± 0.08<br>(0.08-0.31)
GWS	1	0.03	0.33	0.55	5.54	n.d.	0.03	0.06	0.07	0.03	0.07	0.73	2.90	n.d.	0.03
Lebi 2	3	detected[h]<br>(<LOQ-0.03)	0.24 ± 0.02<br>(0.21-0.25)	n.d.	0.04 ± 0.00<br>(0.04-0.04)	n.d.	n.d.	0.15 ± 0.02<br>(0.12-0.17)	0.42 ± 0.02<br>(0.39-0.44)	0.03 ± 0.00<br>(0.02-0.03)	0.19 ± 0.01<br>(0.18-0.20)	1.15 ± 0.15<br>(0.94-1.29)	19.3 ± 1.55<br>(17.2-20.8)	n.d.	0.07 ± 0.01<br>(0.06-0.08)
Malawi x Super Skunk	1	0.03	0.43	n.d.	0.03	n.d.	n.d.	0.26	1.07	0.03	0.11	1.46	11.48	0.24	1.73
OG Kush	3	detected[i]<br>(n.d.-0.02)	0.17 ± 0.02<br>(0.14-0.20)	n.d.	detected[j]<br>(<LOQ-0.03)	n.d.	n.d.	0.04 ± 0.02<br>(0.03-0.06)	detected[k]<br>(<LOQ-0.06)	detected[l]<br>(<LOQ-0.02)	0.08 ± 0.01<br>(0.07-0.10)	0.65 ± 0.07<br>(0.58-0.74)	8.68 ± 3.01<br>(6.15-12.9)	n.d.	detected[m]<br>(<LOQ-0.02)
Pot of Gold	2	0.04, 0.05	0.55, 0.59	0.64, 0.80	9.43, 9.53	<LOQ, 0.03	0.40, 0.44	0.05, 0.06	0.35, 0.38	0.03, 0.02	0.10, 0.08	1.14, 1.39	7.65, 7.44	0.07, 0.09	0.46, 0.49
Pot of Gold nr. 11	1	0.04	0.57	0.61	9.29	0.02	0.40	0.05	0.31	0.03	0.10	1.06	7.8	0.07	0.47
Purple Punch	3	detected[n]<br>(<LOQ-<LOQ)	0.22 ± 0.02<br>(0.19-0.24)	n.d.	0.04 ± 0.00<br>(0.04-0.04)	n.d.	n.d.	0.04 ± 0.00<br>(0.04-0.04)	0.14 ± 0.02<br>(0.13-0.17)	detected[o]<br>(<LOQ-<LOQ)	0.18 ± 0.01<br>(0.17-0.19)	0.59 ± 0.02<br>(0.58-0.61)	16.8 ± 0.48<br>(16.20-16.91)	n.d.	0.06 ± 0.00<br>(0.06-0.07)
Rascal OG	3	detected[p]<br>(<LOQ-0.05)	0.19 ± 0.03	n.d.	0.03 ± 0.00<br>(0.03-0.03)	n.d.	n.d.	0.04 ± 0.01<br>(0.03-0.06)	0.10 ± 0.01<br>(0.07-0.12)	0.06 ± 0.01<br>(0.05-0.08)	0.17 ± 0.01<br>(0.16-0.19)	1.94 ± 0.27<br>(1.64-2.30)	13.38 ± 1.59<br>(11.2-13.8)	n.d.	0.04 ± 0.01<br>(0.03-0.05)
SFV OG	3	detected[q]<br>(<LOQ-0.02)	0.11 ± 0.02<br>(0.09-0.13	n.d.	detected[r]<br>(<LOQ-0.02)	n.d.	n.d.	0.05 ± 0.00<br>(0.05-0.05)	0.23 ± 0.04<br>(0.17-0.27)	0.04 ± 0.00<br>(0.04-0.05)	0.10 ± 0.01<br>(0.08-0.11)	2.10 ± 0.26<br>(1.79-2.42)	9.67 ± 0.89<br>(8.93-10.9)	n.d.	0.03 ± 0.00<br>(0.03-0.03)
Wappa	3	0.04 ± 0.01<br>(0.04-0.05)	0.18 ± 0.01<br>(0.17-0.19)	n.d.	0.03 ± 0.00<br>(0.02-0.03)	n.d.	n.d.	0.05 ± 0.00<br>(0.05-0.05)	0.22 ± 0.01<br>(0.21-0.23)	0.06 ± 0.01<br>(0.05-0.07)	0.16 ± 0.02<br>(0.15-0.19)	1.85 ± 0.19<br>(1.59-2.03)	11.3 ± 0.71<br>(10.6-12.3)	0.05 ± 0.00<br>(0.01-0.05)	0.26 ± 0.01<br>(0.25-0.26)

Analyte detected in a) n = 3 (100%); b) n = 3 (100%); c) n = 3 (100%); d) n = 6 (75%); e) n = 1 (13%); f) n = 8 (100%); g) n = 5 (63%); h) n = 3 (100%); i) n = 2 (67%); j) n = 3 (100%); k) n = 3 (100%); l) n = 3 (100%); m) n = 3 (100%); n) n = 3 (100%); o) n = 3 (100%); p) n = 3 (100%); q) n = 3 (100%); r) n = 3 (100%) of samples

Table S5 Total cannabinoid contents of varieties. CBL and Δ8-THC were not detected in any of the samples. Standard deviation and range are only given, if n ≤ 3. Limit of quantification (LOQ) of all analytes was 0.02% (considering a 50 mg sample extracted with 2 mL MeOH and 1 : 10,000 dilution of the sample). For calculation of means and standard deviations, levels <LOQ were excluded

		**Mean ± standard deviation (range) [%]**						
**Variety**	**n**	**CBC$_{total}$**	**CBD$_{total}$**	**CBDV$_{total}$**	**CBG$_{total}$**	**CBN$_{total}$**	**Δ^9-THC$_{total}$**	**THCV$_{total}$**
Amnesia	1	0.14	0.03	n.d.	0.62	0.17	14.5	0.04
Amnesia S5	3	0.27 ± 0.02<br>(0.25-0.29)	0.04 ± 0.01<br>(0.03-0.04)	n.d.	1.58 ± 0.21<br>(1.29-1.78	n.d.	18.5 ± 1.72<br>(16.1-20.0)	n.d.
Amnesia x SFV	2	0.15, 0.26	0.47, 0.04	n.d.	0.68, 0.80	0.16, 0.20[a]	11.5, 20.8	0.07, 0.11
Big Bud	3	0.11 ± 0.01<br>(0.10-0.13)	0.03 ± 0.00<br>(0.03-0.03)	n.d.	0.35 ± 0.03<br>(0.37-0.31)	0.18 ± 0.02[b]<br>(0.17-0.20)	14.3 ± 1.17<br>(12.9-15.80)	0.06 ± 0.00<br>(0.05-0.07)
Bubba Kush	3	0.17 ± 0.02<br>(0.14-0.19)	0.03 ± 0.02<br>(0.02-0.06)	n.d.	0.15 ± 0.01<br>(0.13-0.17)	0.08 ± 0.01<br>(0.07-0.10)	11.4 ± 1.37<br>(10.2-13.3)	0.04 ± 0.00<br>(0.04-0.05)
C7	1	0.13	0.05	n.d.	0.25	0.24	14.59	0.08
C7 x Thai	1	0.41	5.97	0.04	0.09	0.08	3.23	0.03
Durban x Malawi	8	0.12 ± 0.01<br>(0.10-0.14)	0.04 ± 0.02[c]<br>(0.02-0.04)	n.d.	0.21 ± 0.06<br>(0.14-0.27)	0.14 ± 0.03[d]<br>(0.08-0.17)	14.6 ± 2.05<br>(11.6-17.2)	0.15 ± 0.07[e]<br>(0.07-0.30)
GWS	1	0.32	5.41	0.03	0.12	0.09	3.27	0.02
Lebi 2	3	0.23 ± 0.02[f]<br>(0.21-0.25)	0.04 ± 0.00	n.d.	0.52 ± 0.03	0.20 ± 0.01	18.11 ± 1.35	0.06 ± 0.01
Malawi x Super Skunk	1	0.40	0.03	n.d.	1.20	0.13	11.53	1.75
OG Kush	3	0.15 ± 0.03<br>(0.13-0.19)	0.02[g]<br>(<LOQ-0.02)	n.d.	0.06 ± 0.04[h]<br>(0.03-0.11)	0.08 ± 0.02[i]<br>(0.07-0.11)	8.26 ± 2.71<br>(5.98-12.06)	0.02[j]<br>(<LOQ-0.02)
Pot of Gold	2	0.56, 0.54	8.90, 9.16	0.35[k], 0.41	0.36, 0.40	0.12, 0.09	7.84, 7.92	0.47, 0.52
Pot of Gold nr. 11	1	0.54	8.8	0.36	0.32	0.12	7.90	0.48
Purple Punch	3	0.20 ± 0.02<br>(0.18-0.22)	0.03 ± 0.00<br>(0.03-0.04)	n.d.	0.16 ± 0.02<br>(0.15-0.19)	0.17 ± 0.00[l]<br>(0.16-0.17)	15.4 ± 0.41<br>(14.8-15.8)	0.06 ± 0.00<br>(0.05-0.06)
Rascal OG	3	0.19 ± 0.03<br>(0.16-0.24)	0.03 ± 0.00<br>(0.03-0.03)	n.d.	0.13 ± 0.03<br>(0.09-0.17)	0.21 ± 0.01<br>(0.19-0.22)	13.7 ± 1.65<br>(11.5-15.5)	0.04 ± 0.01<br>(0.03-0.04)
SFV OG	3	0.12 ± 0.01<br>(0.10-0.13)	0.02, 0.02[m]<br>(<LOQ-0.02)	n.d.	0.25 ± 0.03<br>(0.20-0.28)	0.13 ± 0.01<br>(0.11-0.15)	10.6 ± 1.03<br>(9.62-12.00)	0.03 ± 0.00<br>(0.02-0.03)
Wappa	3	0.20 ± 0.01<br>(0.18-0.22)	0.02 ± 0.00<br>(0.02-0.03)	n.d.	0.25 ± 0.01<br>(0.24-0.25)	0.20 ± 0.01<br>(0.19-0.22)	11.7 ± 0.74<br>(11.2-12.8)	0.28 ± 0.01<br>(0.27-0.30)

Additional information on analyte detection: a) CBN <LOQ; b) CBN detected in n = 3 (100%), once < LOQ; c) CBD detected in n = 8 (100%), once <LOQ; d) CBN detected in n = 8 (100%), once <LOQ; e) THCV detected in n = 5 (63%), 4 times <LOQ; f) CBD detected in n = 3 (100%), once <LOQ; g) CBD detected in n = 0 (0%), CBDA detected in n = 3 (100%), twice <LOQ; h) CBG detected in n= 3 (100%), all >LOQ, CBGA detected in n = 3 (100%), twice <LOQ; i) CBN detected in n = 3 (100%), once <LOQ; j) THCV detected in n = 0 (0%), THCVA detected in n = 3 (100%), twice <LOQ; k) CBDV detected <LOQ; l) CBN detected in n = 3 (100%), once < LOQ; m) CBD detected in n = 0 (0%), CBDA detected in n = 3 (100%), once <LOQ. If not noted otherwise, acid forms were detected >LOQ in all samples.

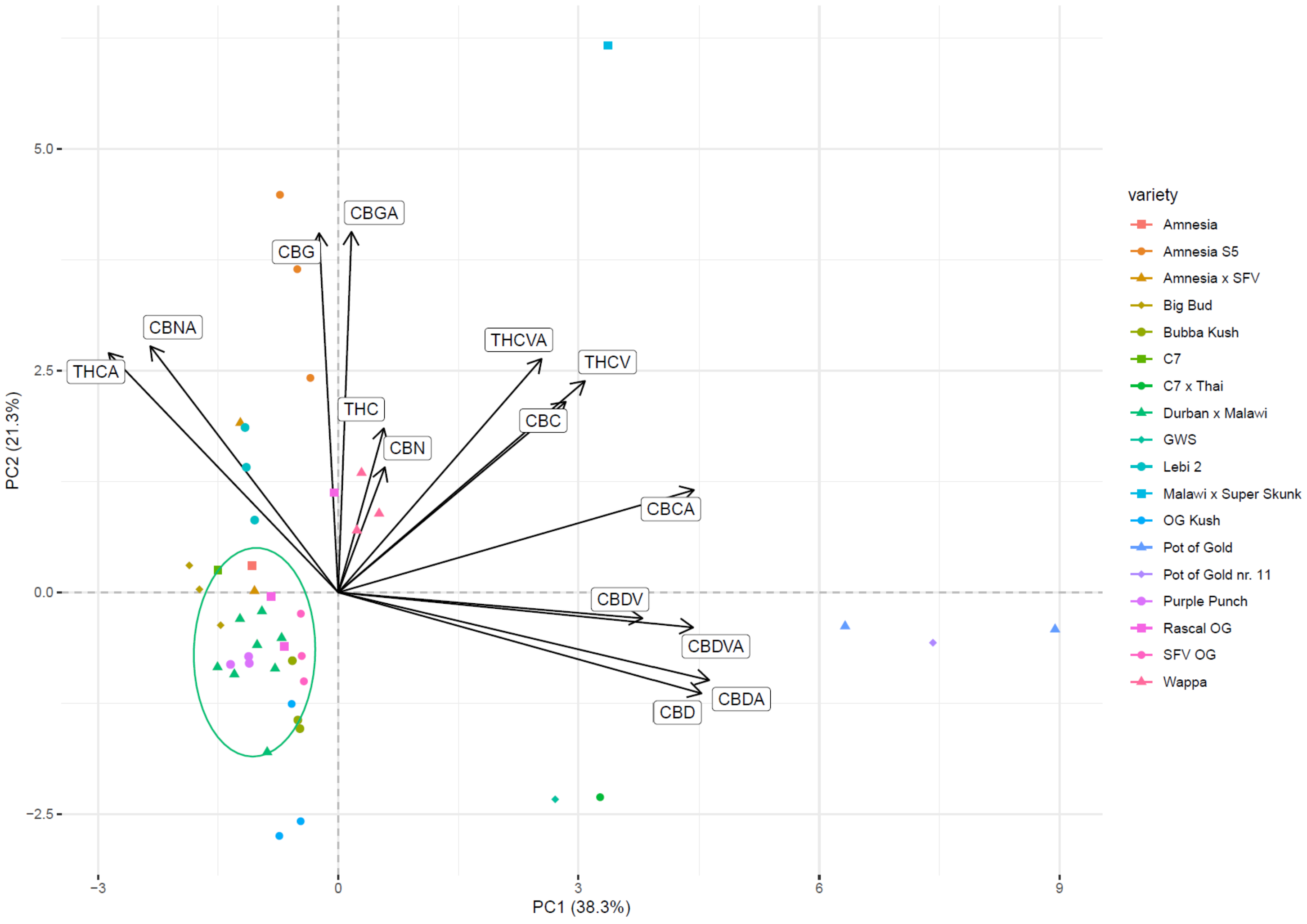

Figure S6 PCA loading plot of the targeted analysis. The 95% confidence interval (95%-CI) is indicated for the variety Durban x Malawi as green ellipse. Due to the limited number of plants per variety (n ≤ 3), the 95%-CI could only be calculated for this variety (n = 8)

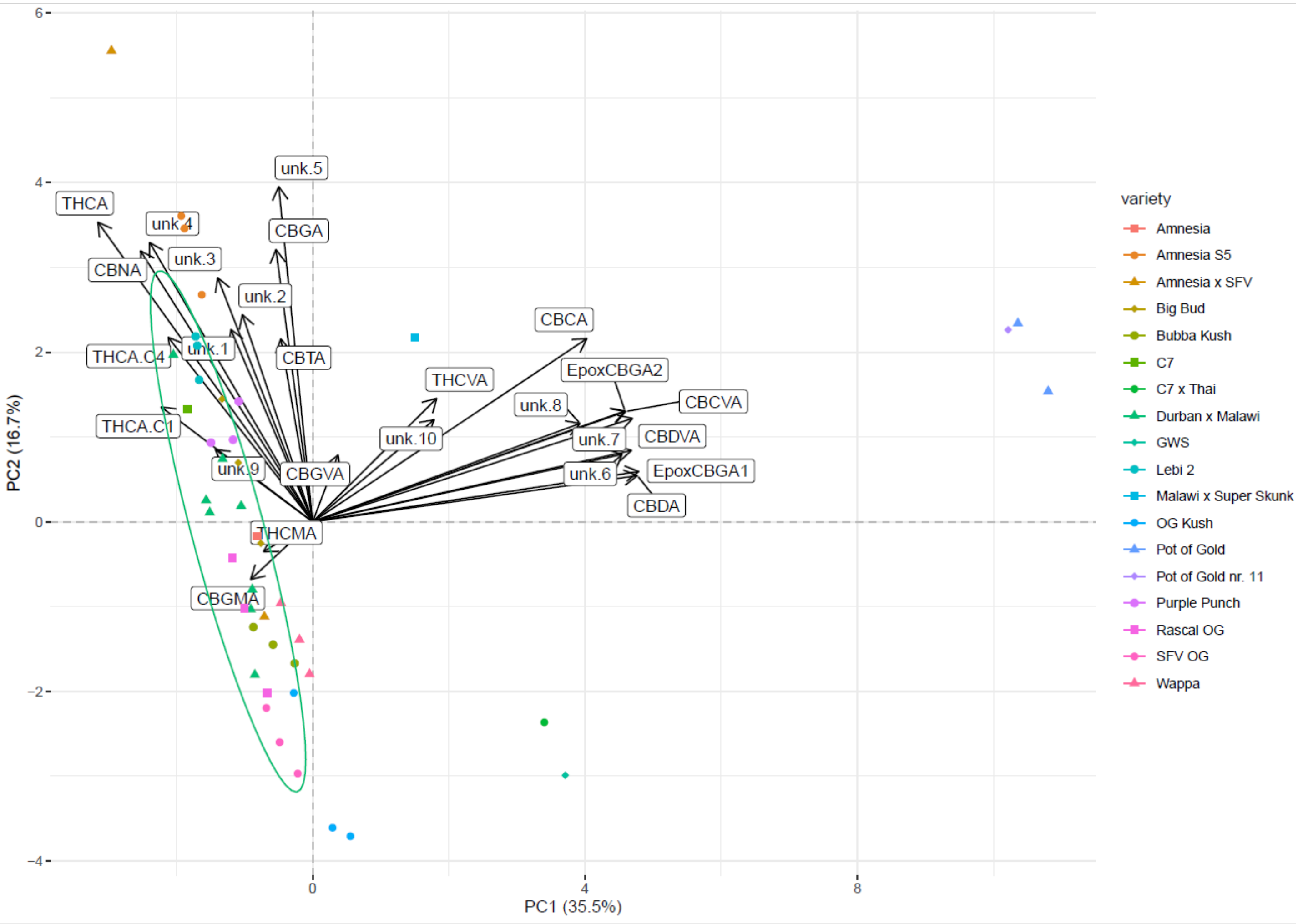

Figure S7 PCA loading plot of the untargeted analysis. The 95% confidence interval (95%-CI) is indicated for the variety Durban x Malawi as green ellipse. Due to the limited number of plants per variety (n ≤ 3), the 95%-CI could only be calculated for this variety (n = 8)

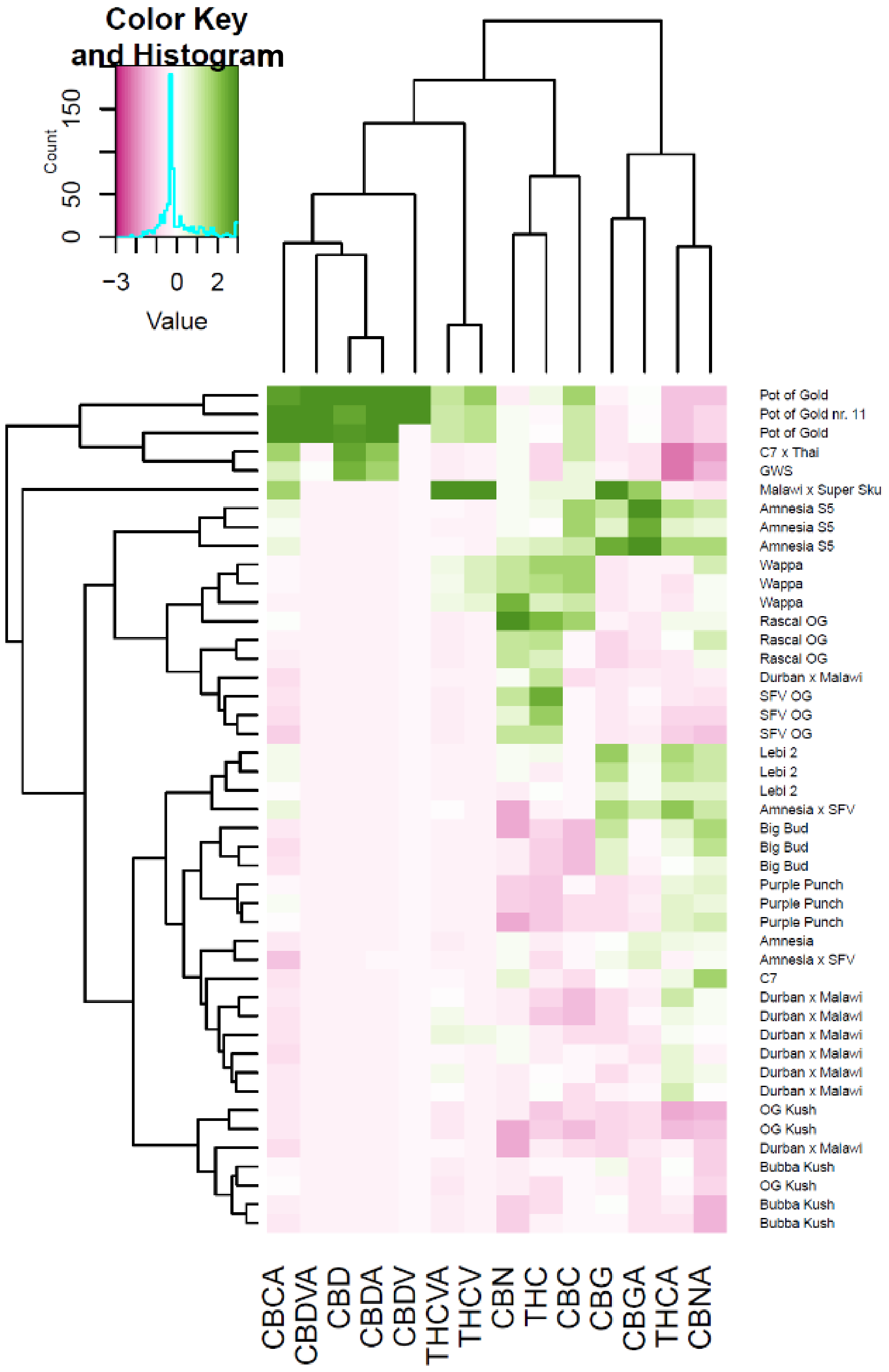

Figure S8 Heatmap applying hierarchical clustering of the targeted data (contents). Green indicates varieties with expressing above-average levels and red indicates where analytes are expressed in below-average levels. Compounds and varieties are sorted in a hierarchical manner, meaning that varieties are sorted based on similar chemical fingerprints, whilst analytes that are typically detected simultaneously are presented side by side. The clustering of the varieties Pot of Gold, Pot of Gold nr. 11, C7 x Thai, and GWS is largely explained by CBCA, CBDVA, CBD, CBDA, and CBDV. Malawi x Super Skunk presents elevated THCV and THCVA levels. Durban x Malawi (grown from seeds) presented six plants that hierarchically clustered close together, while two individual plants are more similar to other plant varieties (e.g., Bubba Kush, and OG Kush for one plant, and SVG OG, Rascal OG for the other). CBGA and CBG are expressed at highest levels for Malawi x Super Skunk and Amnesia S5.

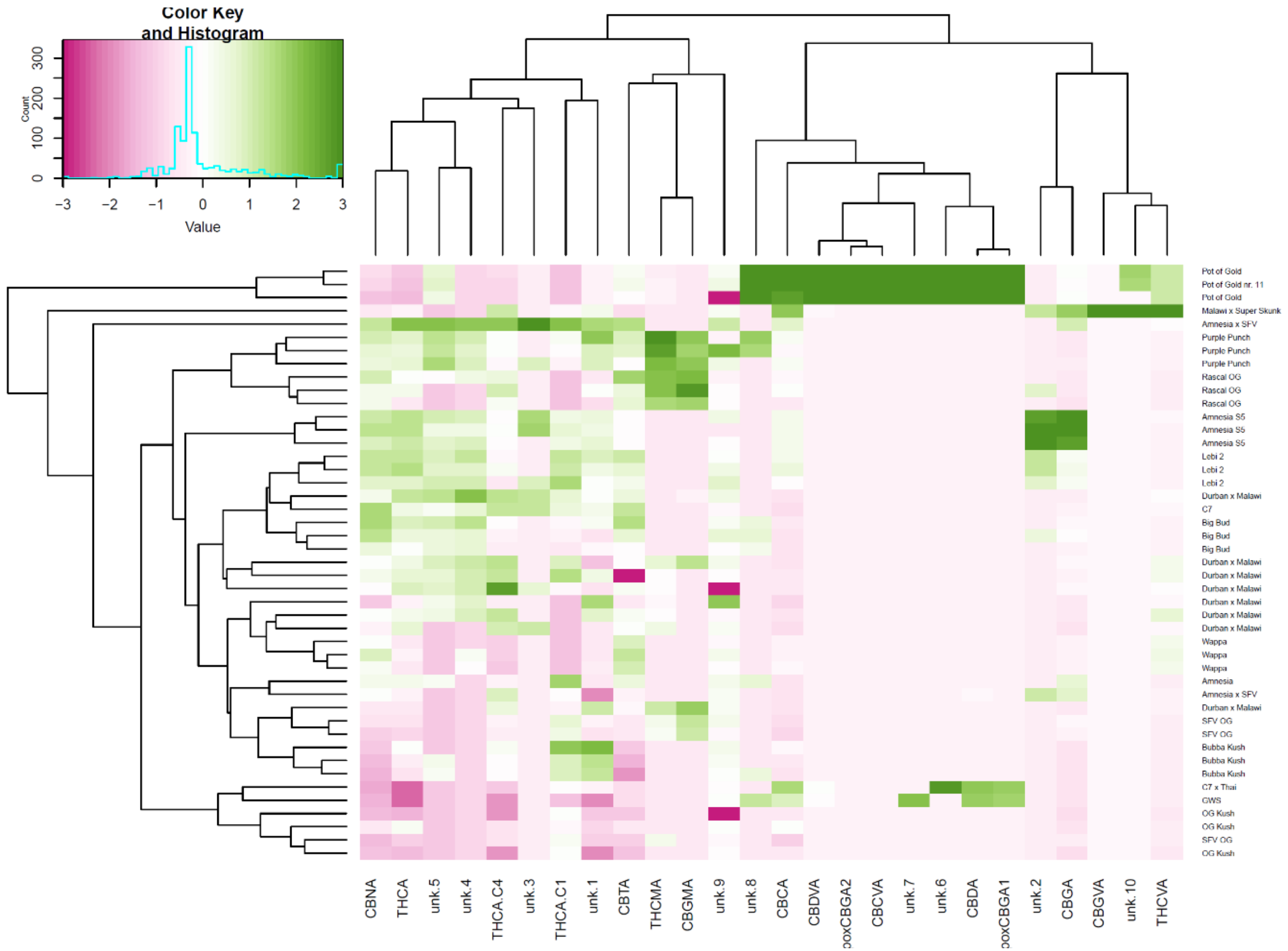

Figure S9 Heatmap applying hierarchical clustering of the untargeted data (weight normalized areas). Green indicates varieties with expressing above-average levels and red indicates where analytes are expressed in below-average levels. Compounds and varieties are sorted in a hierarchical manner, meaning that varieties are sorted based on similar chemical fingerprints, whilst analytes that are typically detected simultaneously are presented side by side. The clustering of the varieties Pot of Gold, Pot of Gold nr. 11, C7 x Thai, and GWS is largely explained by CBDA, CBCA, CBDVA, 6,7-Epoxy-CGA isomers 1 and 2, CBCVA, unknown 6, unknown 7, and unknown 8. Malawi x Super Skunk is characterized by elevated THC and THCV levels. THCMA and CBDMA are highly indicative for the varieties Purple Punch and Rascal OG. Malawi x Super Skunk presents a unique chemical fingerprint largely attributable to the presence of CBGVA, unknown 10, and THCVA.
